# Enhancing anti-tumour immunity through modulating dendritic cell activation by combination therapy with a novel TLR2 agonist and PD-L1 Blockade

**DOI:** 10.1186/s13046-025-03571-9

**Published:** 2025-11-25

**Authors:** Chao Wang, Zhenkang Ai, Yuanhao Wang, Maocai Luo, Tao Wu, Di Wang, Xin Liu, Jiaoyan Lv, Xueheng Guo, Zhisong Wang, Qingqing Sun, Xuebin Liao, Li Wu

**Affiliations:** 1 Institute for Immunology, School of Medicine, Tsinghua University, Beijing, 100084 China; 2 Joint Graduate Program of Peking-Tsinghua-NIBS, School of Life Sciences, Tsinghua University, Beijing, 100084 China; 3 Beijing Key Laboratory of Immunological Research of Allergy, Beijing, 100084 China; 4 School of Pharmaceutical Sciences, Tsinghua University, Beijing, 100084 China; 5 School of Life Sciences, Tsinghua University, Beijing , 100084 China; 6 Tsinghua-Peking Center for Life Sciences, Tsinghua University, 100084 Beijing, China

**Keywords:** Dendritic cell, TLR2, SUP3, PD-L1, Antigen cross-presentation, Tumor immunotherapy

## Abstract

**Background:**

Dendritic cells (DCs) play a predominant role in antitumor immunity. As professional antigen-presenting cells (APCs), DCs can be functionally matured by TLR2 ligand binding to enhance innate immune response and subsequent T cell-dependent adaptive immunity. DC function is often suppressed by the tumor microenvironment, while current TLR2 agonists exhibit suboptimal stability and diminished efficacy in vivo. Therefore, reactivation of suppressed DCs could be a promising strategy for enhancing the efficacy of cancer immunotherapy.

**Methods:**

To investigate the antitumor immunity induced by the novel Toll-like receptor 2 (TLR2) agonist SUP3 with better stability, we established murine melanoma, colon cancer and breast cancer tumor models. The hematopoietic growth factor Flt3L-dependent dendritic cells (FLDCs) were generated and utilized to examine their capacities of antigen processing and cross-presentation, and migration to the tumor-draining lymph nodes (TdLNs) in response to SUP3 treatment. To further improve the antitumor response of SUP3 by increasing the abundance and activation of DCs, Flt3L was administrated in vivo in combination with immune checkpoint blockade.

**Results:**

SUP3 exhibited stronger inhibition of tumor growth and metastasis than classical TLR2 agonist, Pam3. SUP3 could increase cDC1 antigen cross-presentation and TdLN migration, promoting the proliferation, activation and cytotoxicity of antigen-specific cytotoxic T lymphocytes (CTL). SUP3 promoted the intracellular accumulation of antigens and facilitated the process of antigen cross-presentation, the processe regulated by the small GTPase Rab7. SUP3 induced PD-L1 expression by DCs via an interferon-γ-independent pathway. The combination of SUP3 treatment with immune checkpoint blockade by anti-PD-L1 further improved the antitumor response. Moreover, Flt3L increased DC proliferation and infiltration into the tumor tissues that further enhanced the effects of antitumor immunotherapy when used in combination with SUP3 and anti-PD-L1.

**Conclusions:**

This study demonstrated that the modified and more stable TLR2 agonist SUP3 provided an optimal strategy for promoting antitumor immunity via activation of cDC1. SUP3 enhanced antigen cross-presentation by cDC1 and subsequent activation of CTLs. The antitumor effect was further enhanced when SUP3 and Flt3L synergized with PD-L1 blockade. Therefore, reactivation of suppressed DCs in tumor microenvironment would be a promising strategy for designing effective antitumor immunotherapy.

**Supplementary Information:**

The online version contains supplementary material available at 10.1186/s13046-025-03571-9.

## Background

Dendritic cells (DCs) are potent antigen-presenting cells (APCs) that promote antigen-specific cellular immunity [1]. DCs can sense diverse danger signals through different pattern recognition receptors (PRRs) to initiate innate responses and to induce adaptive immunity through antigen presentation. Mature DCs express high levels of costimulatory molecules, cytokines, and chemokines, all contributing to T-cell activation. Mouse DCs consist of three main subsets: the CD11c^+^CD8α^+^ and/or CD103^+^ cDC1 and CD11c^+^CD11b^+^ cDC2 subsets and CD11c^int^SiglecH^+^B220^+^ plasmacytoid DCs (pDCs) [2].

cDC1s are critical for antitumor immunity, as *Batf3*^*–/–*^ mice that are defective in cDC1 development fail to induce T-cell activation and infiltration into spontaneous oncogenic-driven melanoma tissue [3], or to inhibit the growth of transplanted fibrosarcomas [4]. Intratumoral migratory CD103^+^ cDC1s are the major type of APCs that transport tumor antigens to the tumor-draining lymph nodes (TdLNs) to elicit a specific CD8^+^ T-cell response, and this process is CCR7 dependent [5, 6]. However, in the tumor microenvironment (TME), DCs are often under an immune suppressive condition, as DC functions can be affected by tumor cells, leading to immune suppression [7] and the subsequent tumor evasion. In addition, the presentation of tumor-associated antigens (TAAs) in the absence of costimulatory signals leads to T-cell anergy, and the induction of inhibitory molecules, e.g. PD-1 and CTLA-4 etc., can curb T-cell effector activity [8]. Usually, very few cDC1s can be detected in the TME owing to reduced recruitment or differentiation via various mechanisms, including the production of immunosuppressive factors, cytokines, and chemokines [9, 10]. However, it has been shown that the abundance/density of tumor-infiltrating DCs directly associated with the outcomes of the antitumor immune response. Programmed cell death 1 ligand 1 (PD-L1) expression on DCs and other cells in the TME also inhibits T-cell proliferation and cytokine production by PD-1-expressing T cells [11, 12].

Enhancing the effectiveness of anticancer immunotherapy through modulation of DC functions in TME has become a major focus of cancer immunology research. Various approaches have been proposed to target DCs, including the use of immune modulators and antigens to mobilize and activate endogenous DCs, as well as DC-based tumor vaccines [13, 14]. A comprehensive understanding of the diversity and functions of DC subsets in the TME, and the mechanisms by which the TME shapes DC activity, may facilitate the development of a more effective strategy for cancer immunotherapy.

Previously we reported a novel TLR2 agonist SUP3, optimized from Pam3 with enhanced stability and activation [15]. In this study, we determined the function of SUP3, and further tested the effects of combination therapy of SUP3 with anti-PD-L1 and Flt3L, a DC promoting cytokine, in mouse tumor models. Our findings revealed that SUP3 could intensify antitumor immunity by stimulating DC activation, especially cDC1 activation in the TME, and augmenting antitumor-specific T-cell responses. We also demonstrated that the antitumor immunity of SUP3 could be further synergized by anti-PD-L1 therapy, and combination with Flt3L treatment, resulted in significantly more effective eradication of tumor growth.

## Methods

### Tumor model and treatment

A total of 1 × 10^6^ MC38 or 4T1 tumor cells were subcutaneously inoculated into the right flank of mice. When the tumor reached a certain volume (70–100 mm^3^), groups of mice were treated with SUP3 or Pam3CSK4 (Invivogen) paratumorally every other day. A total of 5 × 10^5^ B16F10, B16F10-RFP (B16F10 is engineered to stably express red fluorescent protein) or B16F10-Flt3L (B16F10 is engineered to stably express Flt3L) tumor cells were subcutaneously injected into the right flank of mice when the tumor reached a certain volume (70–100 mm^3^). The mice were treated with SUP3 or Pam3CSK4 paratumorally once every two days, for a total of 4 times, unless otherwise noted. For antibody treatment, mice were treated with 150 µg PD-L1 antibody (BioXCell, RRID: AB_ 10949073) intraperitoneally twice a week. Groups of mice were treated with 200 µg anti-CD4 mAb, 200 µg anti-CD8 mAb, 200 µg anti-NK1.1 mAb or 200 µg IgG control intraperitoneally on days 0, 3, and 7, followed by a 200 µg dose every 7 days. The in vivo neutralizing antibodies above were purchased from BioXCell company. For Flt3L administration, mice were injected with 30 µg Flt3L (BioXCell) or control PBS intraperitoneally for nine consecutive days. To inhibit T-cell trafficking, mice were injected intraperitoneally with 20 µg FTY720 (Sigma) every day at the indicated time. Tumor volume was calculated from length (L) and width (W) using the formula: tumor volume = L×W×W/2.

### In vitro generation of bone marrow-derived DCs and DC adoptive transfer

Wild-type (WT) or *Tlr2*^–/–^ mouse bone marrow cells were collected, erythrocytes lysed with red cell removing buffer (RCRB), and single mouse bone marrow cells were cultured in RPMI 1640 with 10% fetal bovine serum supplemented with 200 ng/mL Flt3L (PeproTech) and *β*-mercaptoethanol (referred to as FLDCs). For FLDC analysis, the CD11c^+^ DC subsets SiglecH^+^ pDCs, CD24^+^ cDC1s (referred to as FLDC1s) and CD172^+^ cDC2s (referred to as FLDC2s) were sorted after 7 days. For DC adoptive transfer, C57BL/6 mice were inoculated with 5 × 10^5^ B16F10 tumor cells, and FLDC1s (2–4 × 10^7^/mL) were treated with SUP3 (1 µM) for 4 h or subjected to silencing with a *Rab7* siRNA kit (GenePharma) for at least 24 h and then incubated with 50 µg/mL recombinant TRP2 (GenScript) for 4 h. cDC1s (1–2 × 10^6^) were washed with cold PBS and adoptively transferred into mice intravenously on days 3 and 7 after tumor inoculation.

### DC antigen cross-presentation

Flt3L cultured bone marrow-derived cDC1s were pretreated with SUP3 (1 µM) for 4 hours and then pulsed with OVA (50 µg/mL, Sigma) or SIINFEKEL (1 ng/mL, eBioscience) for 4 hours. Excess antigen was washed with cold PBS, and antigen-specific OT-I CD8^+^ T cells were enriched with a negative selection cocktail: anti-CD11b (M1/7), anti-F4/80 (F4/80), anti-TER119 (TER-119), anti-Gr1 (RB6-8C5), anti-MHCII (M5/114), anti-CD19 (ID3), anti-B220 (RA36B2), and anti-CD4 (GK1.5). Purified CD8^+^ T cells (1 × 10^7^/mL) were labeled with 5 µM CFSE for 10 min at 37 °C. A total of 1–2 × 10^4^ cDC1s were cocultured with 1 × 10^5^ CD8^+^ T cells. T-cell proliferation was analyzed by flow cytometry after 72 h. For the in vivo assay, tumor-infiltrating CD103^+^ cDC1s were sorted from B16F10-OVA tumor-bearing mice, and purified CD103^+^ cDC1s were treated with SUP3 (1 µM) and/or anti-PD-L1 Abs (1 µg/mL) for 4 hours. OT-I mouse-derived CD8^+^ T cells were enriched as previously noted. A total of 2 × 10^4^ cDC1s were cocultured with 1 × 10^5^ CD8^+^ T cells in a 96-well plate. T-cell proliferation was analyzed by flow cytometry after 72 hours.

### Antigen uptake and deposition by DCs and detection of endosomal pH

Flt3L cultured bone marrow-derived cDC1s were pretreated with SUP3 (1 µM) for 4 h and then pulsed with OVA-AF488 (5 µg/ml, Invitrogen) for 30 min at 37 °C or on ice as a control. Antigen uptake was measured by flow cytometry. For antigen deposition, cDC1s were pretreated with SUP3 (0.5–1.5 µM) for 4 h and then pulsed with OVA-AF488 (5 µg/ml) for 30 min at 37 °C. Cellular fluorescence intensity was measured at the indicated time points (15, 30, 60, and 120 min), and cells were washed with cold PBS twice, fixed with 2% PFA, and then mounted with DAPI. Cell images were captured by confocal microscopy (TCS SP8 gSTED 3X, Leica). For endosomal pH detection, cDC1s were pretreated with SUP3 (0.5–1.5 µM) for 4 h, pulsed with FITC-dextran (1 mg/mL, MCE) for 15 min at 37 °C. The procedure was then stopped, and the cells washed with cold PBS-1% FBS. The mean florourescence intensity (MFI) was analyzed by flow cytometry at the indicated time points. The detected pH was normalized to a standard curve generated from cells pulsed in solutions with different pH ranging from 4 to 8.

### In vivo DC migration

Flt3L cultured cDC1s were generated and pretreated with SUP3 (1 µM) or PBS for 4 h. Then, treated and untreated cells were differentially labeled with CFSE (5 µM, Invitrogen) or CTV (5 µM, Invitrogen) for 10 min at 37 °C. The cells were mixed at a 1:1 ratio and assessed by flow cytometry before adoptive transfer. A total of 2 × 10^6^ labeled DCs were injected into the vein of the footpad of CD45.1^+^ recipient mice. Inguinal lymph nodes were obtained and digested with collagenase and DNase, and migrated DCs were measured by flow cytometry after 16 h.

### Quantification and statistical analysis

All data are presented as the mean ± SEM. Two-way ANOVA or an unpaired two-tailed Student’s *t* test was used. For the mouse survival study, the log-rank (Mantel‒Cox) test was performed. For the tumor growth study, the results were subjected to endpoint analysis when two groups were compared or to two-way ANOVA when multiple groups were compared. Experiments were repeated two to three times. Data were analyzed and presented with Prism 8.0 software (GraphPad). *p* < 0.05 was considered significant: *p < 0.05, **p < 0.01, ***p < 0.001, and ****p < 0.0001.

## Results

### SUP3 exerted an enhanced antitumor effect in vivo

**Fig. 1:**
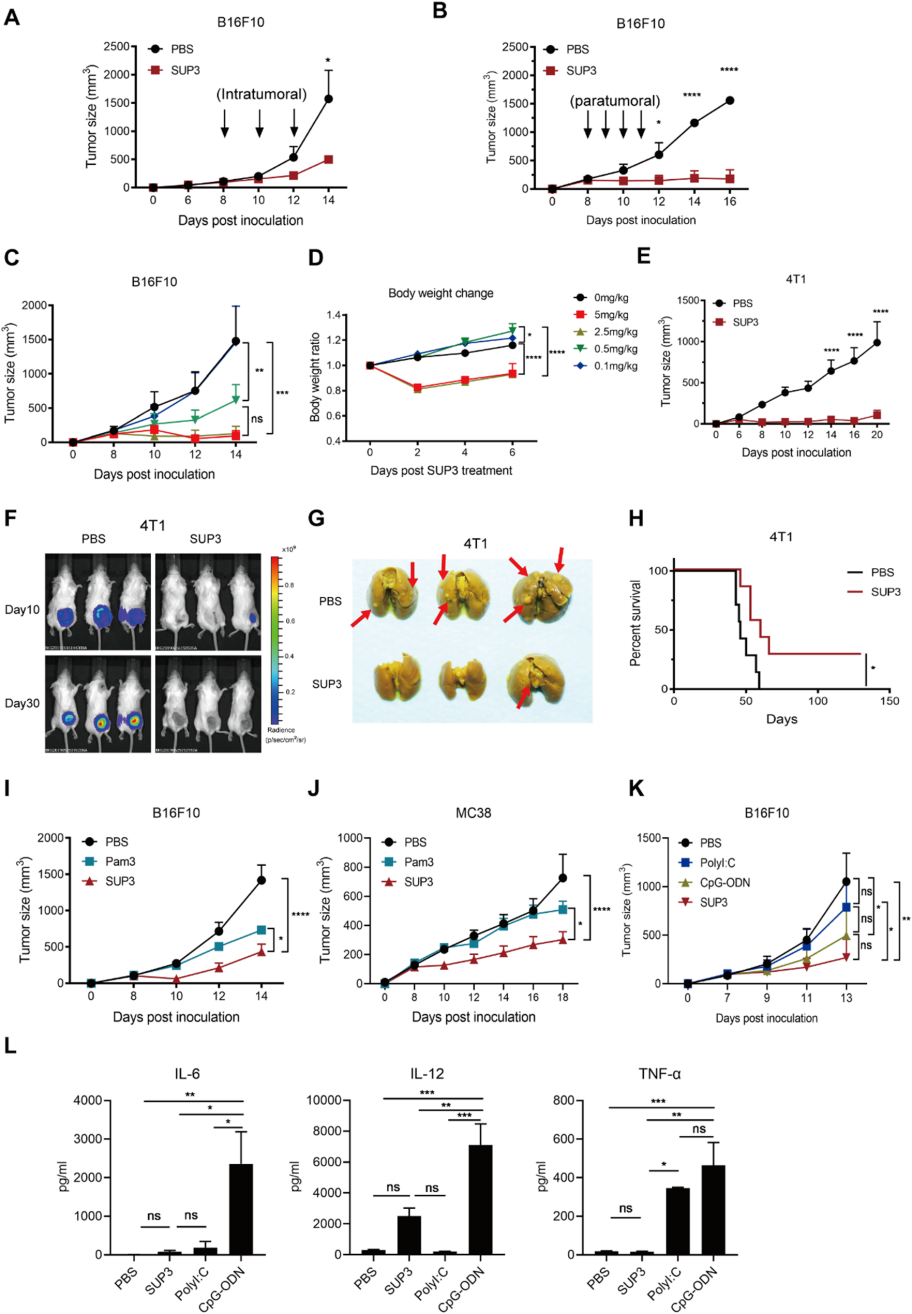
SUP3 inhibited tumor growth in mice.**A** B16F10 tumor-bearing mice were treated with intratumoral injections of SUP3 (5 mg/kg) on days 8, 10, and 12 after tumor challenge. *n* = 5 per group. **B** B16F10 tumor-bearing mice were treated with paratumoral injections of SUP3 (0.5 mg/kg) on days 8, 9, 10, and 11 after tumor challenge. The mean ± SEM of two independent experiments is shown; *n* = 5. **C**, **D** B16F10 tumor-bearing mice were treated with paratumoral injections of SUP3 (0, 0.1, 0.5, 2.5, and 5 mg/kg) on days 8, 10, and 12 after tumor challenge. Tumor size and the body weight ratio were measured; *n* = 3–5. In the 0 mg/kg or PBS group, the mice were treated with saline. **E** 4T1 breast cancer-bearing mice were treated (s.c.) with a single dosage of SUP3 paratumorally on day 6 post-tumor inoculation, and tumor growth was measured; *n* = 7. **F**, **G** 4T1 breast cancer-bearing mice were treated (s.c.) with a single dosage of SUP3 paratumorally on day 6 post-tumor inoculation. Mouse primary tumors and lung metastatic lesions were detected by IVIS 4 weeks later; *n* = 5. **H** Survival curves of 4T1 tumor-bearing mice were generated; *n* = 7. (I) B16F10 tumor-bearing mice were paratumorally injected with SUP3 or Pam3CSK4 (0.5 mg/kg) on days 7, 9, 11, and 13 after tumor challenge, and tumor size was determined; *n* = 5 per group. **J** MC38 tumor-bearing mice were treated with paratumorally injected SUP3 (0.5 mg/kg) or Pam3CSK4 (0.5 mg/kg) on days 7, 9, 11, and 13 after tumor challenge; *n* = 5. **K** B16F10 tumor-bearing mice were treated with paratumorally injected SUP3, Poly I: C or CpG-ODN (0.5 mg/kg) on days 7, 9, 11, and 13 after tumor challenge, and serum cytokines (IL-6, IL-12 and TNF-α) were detected; *n* = 5 per group **L**. Data were analyzed by two-way ANOVA (A-E, I-L) and the log-rank (Mantel‒Cox) test (H). ns, not statistically significant; *P < 0.05, **P < 0.01, ****P < 0.0001

Previously, our study showed that TLR2 is predominantly expressed on mouse myeloid cells, with higher expression by DCs and moderate levels by macrophages, monocytes and neutrophils (Supplementary Fig. S1A). High levels of TLR2 expression are positively associated with the overall survival rate in melanoma patients (Supplementary Fig. S1B). In this study, we aimed to evaluate the therapeutic potential of the novel TLR2 agonist in B16F10 tumor-bearing mice. Intratumoral or paratumoral administration of SUP3 8 days after tumor inoculation significantly suppressed tumor growth (Fig. 1A and B). The data demonstrate that peritumoral administration of SUP3 yielded superior efficacy compared to intratumoral administration by Day 14. The antitumor response induced by SUP3 exhibited a dose-dependent effect (Fig. 1C); however, the relatively higher dose (2.5–5.0.5.0 mg/kg) of SUP3 may induce side effects on the host, as reflected by body weight changes (Fig. 1D). Therefore, we selected a modest dose of 0.5 mg/kg administered via paratumoral injection as the optimal dosage for the remainder of this study. Furthermore, administration of 0.5 mg/kg SUP3 did not elicit any pathological changes in the tested organs of the healthy recipient mice, as demonstrated by weight changes, biochemical functional tests (Supplementary Fig S1C and D).

The same phenomenon was observed in the 4T1 metastatic breast tumor model, in which SUP3 significantly suppressed tumor growth (Fig. 1E and F). Notably, distant tumor cell metastasis to the lung was also effectively diminished (Fig. 1G; Supplementary Fig S1F), leading to an improvement in mouse survival rate (Fig. 1H). In the metastatic melanoma model, SUP3 treatment also dramatically suppressed metastasis formation (Supplementary Fig S2A, B).

To determine whether SUP3 has a more potent antitumor effect compared to the traditional TLR2 agonist Pam3CSK4 (Pam3), tumor-bearing mice were treated with SUP3 or Pam3 respectively. We observed that SUP3 treatment resulted in more significant inhibition of tumor growth compared to that of the Pam3 treatment in the B16F10 tumor model (Fig. 1I). Furthermore, in the MC38 colon cancer model, SUP3 demonstrated superior tumor growth inhibition compared to Pam3 (Fig. 1J). We also compared the antitumor effects of SUP3 with other TLR agonists in B16F10 tumor-bearing mice treated with the TLR3 agonist PolyI: C and the TLR9 agonist CpG-ODN. Although it did not reach statistical significance, the results may imply that SUP3 had better antitumor effects (Fig. 1K). In addition, we collected mouse serum after 4 h and found that PolyI: C and CpG-ODN induced the release of high levels of cytokines such as IL-6, IL-12, and TNF-α. However, SUP3 only induced low levels of IL-12 expression (Fig. 1L). Moreover, administration of 0.5 mg/kg SUP3 did not trigger a significant increase in systemic levels of proinflammatory cytokines (TNF-α, and IFN-β) compared to the LPS control (Supplementary Fig. S1E). As SUP3 is derived from Pam3 through structural modification, these data indicated that SUP3 might be a promising candidate for developing TLR2-based antitumor therapies.

### The enhanced antitumor effects of SUP3 were mediated by adaptive T-cell immune responses

**Fig. 2:**
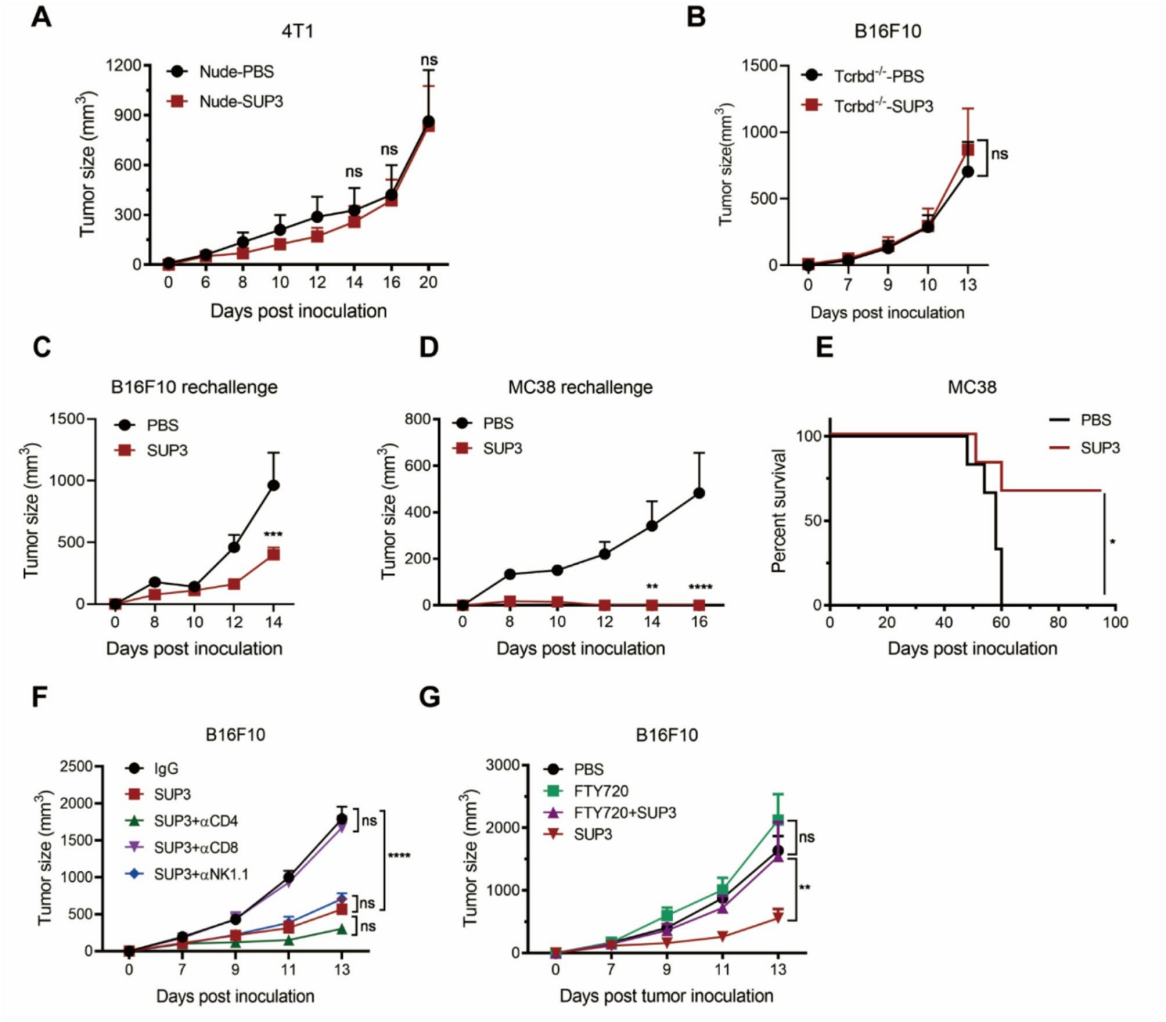
The effects of SUP3 were mediated by adaptive T-cell immune responses (**A**) BALB/c nude mice were inoculated with 4T1 tumor cells and treated with SUP3 (0.5 mg/kg), and the tumor size was measured; *n* = 5. **B** Tcrbd^−/−^ mice were inoculated with B16F10 tumor cells and treated with SUP3 (0.5 mg/kg), and the tumor size was measured; *n* = 5. **C** B16F10 tumor-bearing mice were treated with SUP3 (0.5 mg/kg) and underwent tumor removal surgery. The mice were rechallenged with tumor cells after 4 weeks and tumor size was measured. *n* = 3 (PBS), *n* = 4 (SUP3). **D** MC38 tumor-bearing mice were treated with paratumorally injected SUP3, MC38 tumor cells were used to rechallenge the mice after 80 days, and tumor size was measured; *n* = 6 per group. **E** The survival rates of rechallenged MC38 tumor-bearing mice were measured, *n* = 6–7. **F** B16F10 tumor-bearing mice were treated with SUP3 (0.5 mg/kg) and/or 200 µg anti-CD4 mAb, anti-CD8 mAb, anti-NK1.1 mAb, or normal IgG as a control, injected intraperitoneally on days 0, 3, and 7, followed by a 200 µg dose every 7 days. Tumor size was measured; *n* = 5. **G** B16F10 tumor-bearing mice were treated with SUP3 (0.5 mg/kg) and/or FTY720 (20 µg/mouse). Tumor size was measured; *n* = 5. **A**-**D**, **F** and **G.** Data are shown as the mean ± SEM. **A**-**G** Results are from 2 independent experiments. Data were analyzed by two-way ANOVA **A**-**D**, **F** and **G** and the log-rank (Mantel‒Cox) test **E**. *ns* not statistically significant, *P < 0.05, **P < 0.01, ***P < 0.001, ****P < 0.0001

To determine which cell type mediated the enhanced anti-tumor effects of SUP3, various mouse models with defects or deletion of different immune cells were tested. SUP3 did not induce an antitumor response in 4T1 tumor-bearing nude mice, which lack T-cell-mediated adaptive immunity, indicating that the antitumor effect of SUP3 is T-cell dependent (Fig. 2A). Moreover, in a Tcrbd^−/−^ mouse tumor model, which exhibited T-cell deficiency, administration of SUP3 did not result in a significant anti-tumor effect in B16F10 tumor-bearing mice (Fig. 2B). These findings suggested that SUP3, as a TLR2 ligand, played an important role in the T-cell-mediated antitumor response.

Notably, 7 days after SUP3 treatment, surgical excision of tumor tissue in B16F10 tumor-bearing mice and reinjection with tumor cells after 30 days, the effect of SUP3 treatment continued to suppress tumor growth. Similarly, MC38 tumor bearing mice were treated with SUP3 and the tumors were removed 14 days later. After 4 weeks these mice were reinoculated with either B16F10 or MC38 tumor cells, significant inhibition of tumor growth was observed (Fig. 2C and D). Moreover, after 80 days of reinoculation, an increased survival rate was observed in SUP3 treated MC38 tumor model (Fig. 2E). These findings suggested that SUP3 treatment might have induced antitumor immune memory response in the host. Also, SUP3 treatment inhibited the B16F10 tumor cell metastasis to lung tissue (Supplementary Fig. S2A, B). In the MMTV-PyMT spontaneous breast cancer model, SUP3 treatment at 10 weeks enhanced tumor infiltration of CD8^+^ T cells, reduced levels of tumor-associated indexes: CD31 and Ki67 (Supplementary Fig. S2C, D), and increased infiltration of CD103^+^ cDC1s (Supplementary Fig S2E, F), which may contribute to antitumor T cell responses. Further more, depletion of natural killer (NK) cells or CD4 + T cells in the SUP3-treated mice did not inhibit tumor growth in the B16F10 melanoma model, indicating that NK cells or CD4^+^ T cells were not the major cell types responsible for the SUP3-mediated antitumor response. In contrast, the antitumor response was diminished when CD8^+^ T cells were depleted (Fig. 2F). Furthermore, when T-cell egress from the draining lymph node was blocked by the injection of the S1P receptor antagonist FTY-720, remarkable tumor growth in mice was observed even treated with SUP3 (Fig. 2G). These results indicated that the enhanced antitumor effects of SUP3 are CD8^+^ T-cell dependent.

### SUP3 enhanced antigen cross-presentation by cDC1s and elicited antigen-specific cytotoxic T-cell responses

**Fig. 3:**
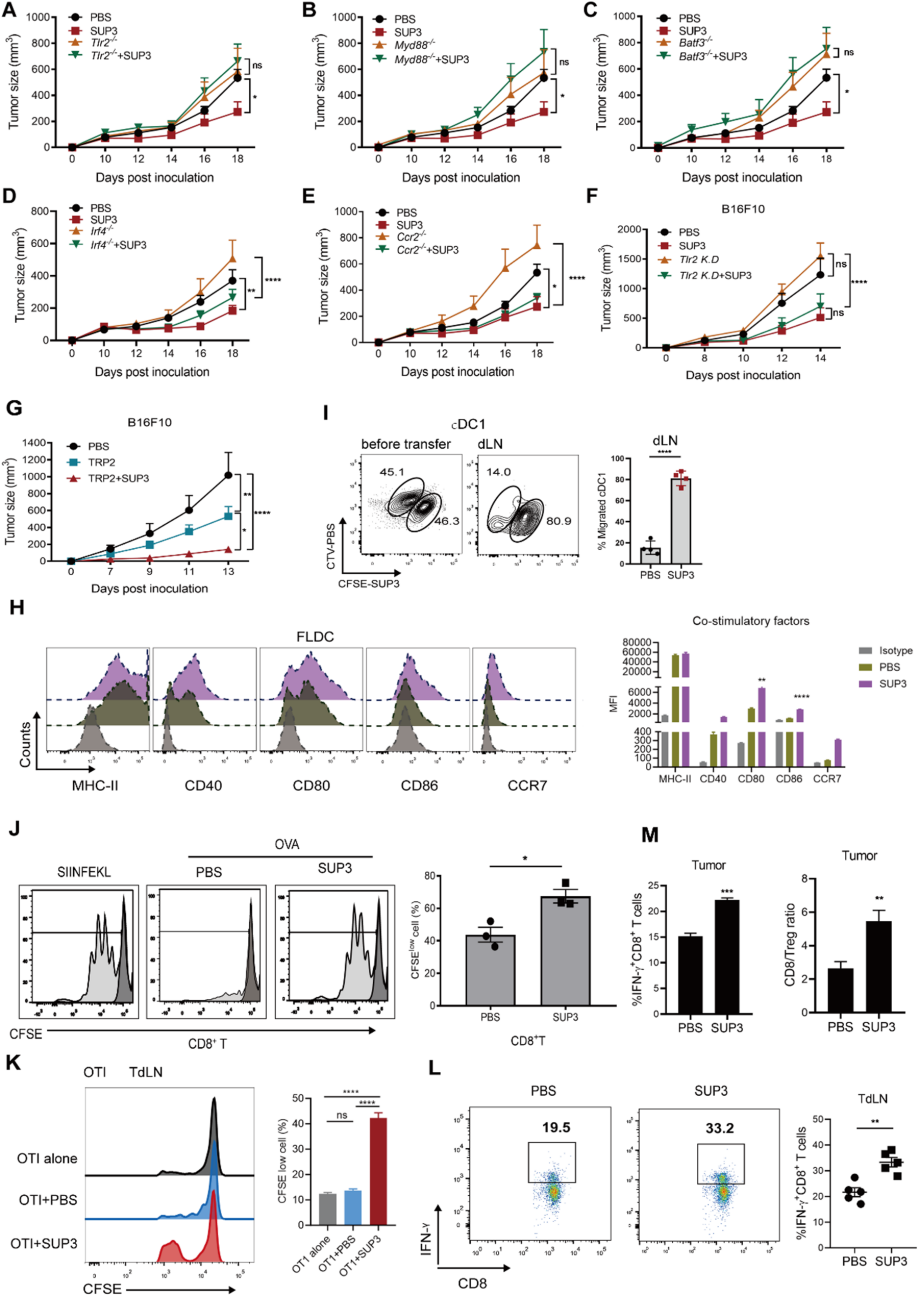
The antitumor effect of SUP3 required cDC1-mediated antigen cross-presentation and T lymphocyte cytotoxicity.**A**-**E** Bone marrow cells from CD45.2 *Tlr2*^–/–^, *Myd88*^–/^, *Batf3*^–/–^ *Irf4*^–/–^ or *Ccr2*^–/–^mice were adoptively transferred into irradiated CD45.1^+^ recipient mice respectively. Tumor-bearing mice were treated with SUP3 (0.5 mg/kg) or PBS, and tumor size was measured dynamically. *n* = 6 per group. **F** TLR2 knockdown B16F10 tumor cells (*Tlr2* K. D) were obtained using the CRISPR‒Cas9 method, and C57BL/6 wild-type mice were inoculated with tumor cells. Tumor-bearing mice were treated with SUP3 (0.5 mg/kg), and tumor size was measured; *n* = 6–7. **G** TRP2-pulsed cDC1s, which were pretreated with SUP3 (1 µM), were injected into B16F10 tumor-bearing mice via the footpad on days 3 and 7 after tumor inoculation, and tumor size was measured; *n* = 6.** H** FLDCs were stimulated with SUP3 (1 µM), and costimulatory molecules, including CD40, CD80, CD86, and MHC-II, were detected after 24 h. **I** The detection of the migration of cDC1s after SUP3 treatment in vivo. **J** T-cell proliferation in vivo was detected by flow cytometry. **K** The proliferation of antigen-specific T-cell in tumor-draining lymph node (TdLN) was assessed after 72 h. OT-I alone group: CFSE-labeled OT-I T cells were adoptively transferred into non-tumor bearing mice; *n* = 3. **L** TdLN CD8^+^ T cells were stimulated with PMA, ionomycin and BFA for 5 h, and IFN-γ^+^ CD8^+^ T cells were measured. **M** Additionally, the ratio of IFN-γ^+^ CD8^+^ T cells to tumor-infiltrating Treg cells was measured. **B**-**I**, **J**-**M** Data are shown as the mean ± SEM. **A**-**M** Results are from 2 to 3 independent experiments. Data were analyzed by two-way ANOVA **A**-**G** and Student’s unpaired *t* test **I**, **J**, **L**, **M**. *ns* not statistically significant, *P < 0.05, **P < 0.01, ***P < 0.001, ****P < 0.0001

Previously, we found that SUP3 did not induce apoptosis or inhibit proliferation of tumor cells in vitro (Supplementary Fig S3A, B). Therefore, we speculated that SUP3 repressed tumor growth via TLR2 signaling by the immune cells of the host. Consistence with this hypothesis we observed that SUP3 treatment no longer showed antitumor activity in *Tlr2*^*–/–*^ or *Myd88*^*–/–*^ bone marrow chimeric mice (Fig. 3A, B). Meanwhile, we also constructed Batf3^–/–^ bone marrow chimeric mice, as the development of CD103^+^ cDC1 was impaired in transcription factor BATF3 deficient mice [4], and we observed that the antitumor effect of SUP3 also diminished, and the tumor continued to grow in those chimeric mice (Fig. 3C). Moreover, we created Ccr2^–/–^ or Irf4^–/–^ bone marrow chimeric mice to test if other myeloid cells, such as macrophages or cDC2s were also involved, and we discovered that the antitumor effect of SUP3 was not affected in those bone marrow chimeric mice (Fig. 3D and E). Although the tumor cells universally expressed various levels of TLR2 (Supplementary Fig S3C), knockdown of TLR2 expression in B16F10 tumor cells did not affect the antitumor response in SUP3-treated mice (Fig. 3F). Additionally, SUP3-pretreated cDC1s that were pulsed with the melanoma cell-specific tumor antigen tyrosinase-related protein 2 (TRP2) demonstrated a robust antitumor response when adoptively transferred into B16F10 tumor-bearing mice (Fig. 3G).

Furthermore, we observed that knockout TLR2 in DC progenitor cells such Lin^–^Sca-1^+^c-Kit^+^ (LSK), common lymphoid progenitor (CLP), common myeloid progenitor (CMP), CD115^+^ common dendritic cell progenitor (CDP) or CD115– CDP cells had no impact on their survival and numbers (Supplementary Fig S4A and B). Additionally, in *Tlr2*^–/–^ MMTV-PyMT mice with spontaneous breast tumors and *Tlr2*^*–/–*^ B16F10 tumor-bearing mice, the infiltrations of CD11b^+^ cDC2s and CD103^+^ cDC1s in tumors were comparable to that of WT counterparts (Supplementary Fig S4C and D). Tumor-infiltrating DCs, especially cDC1s, play an important role in tumor immunotherapy [4, 16]. Therefore, we investigated whether SUP3 enhanced DC function to initiate antitumor immunity. Our results showed that SUP3 promoted maturation of the immature DCs, by upregulating the expression levels of CD40, CD80, CD86, and CCR7 (Fig. 3H). CCR7 expressed on CD103^+^ cDC1s is essential for tumor antigen drainage and T-cell activation [5]. Interestingly, SUP3 promoted cDC1 migration to the lymph nodes when cDC1s were adoptively transferred into recipient mice after 16 h (Fig. 3I). Moreover, SUP3 pretreatment of cDC1s pulsed with OVA induced dramatically enhanced antigen cross-presentation, resulting in the proliferation of OT-I CD8^+^ T cells specific for the antigen (Fig. 3J). In the OVA-expressing B16F10 (B16F10-OVA) tumor model, CFSE-labeled OT-I-positive CD8^+^ T cells were adoptively transferred into B16F10-OVA tumor inoculated mice, and 72 h later increased proliferation of OT-I cells in the tumor-draining lymph nodes (TdLNs) was observed in the SUP3-treated group compared to the control group (Fig. 3K). SUP3 also promoted the secretion of IFN-γ and increased ratio of CD8^+^ T-cell-to-Treg cells (Fig. 3L and M). Collectively, these data revealed that enhanced ability of antigen cross presentation of CD103^+^ cDC1s, and the subsequent CD8^+^ T cell activation were responsible for the antitumor effect of SUP3.

### SUP3 prolonged cDC1 intracellular antigen deposition

**Fig. 4:**
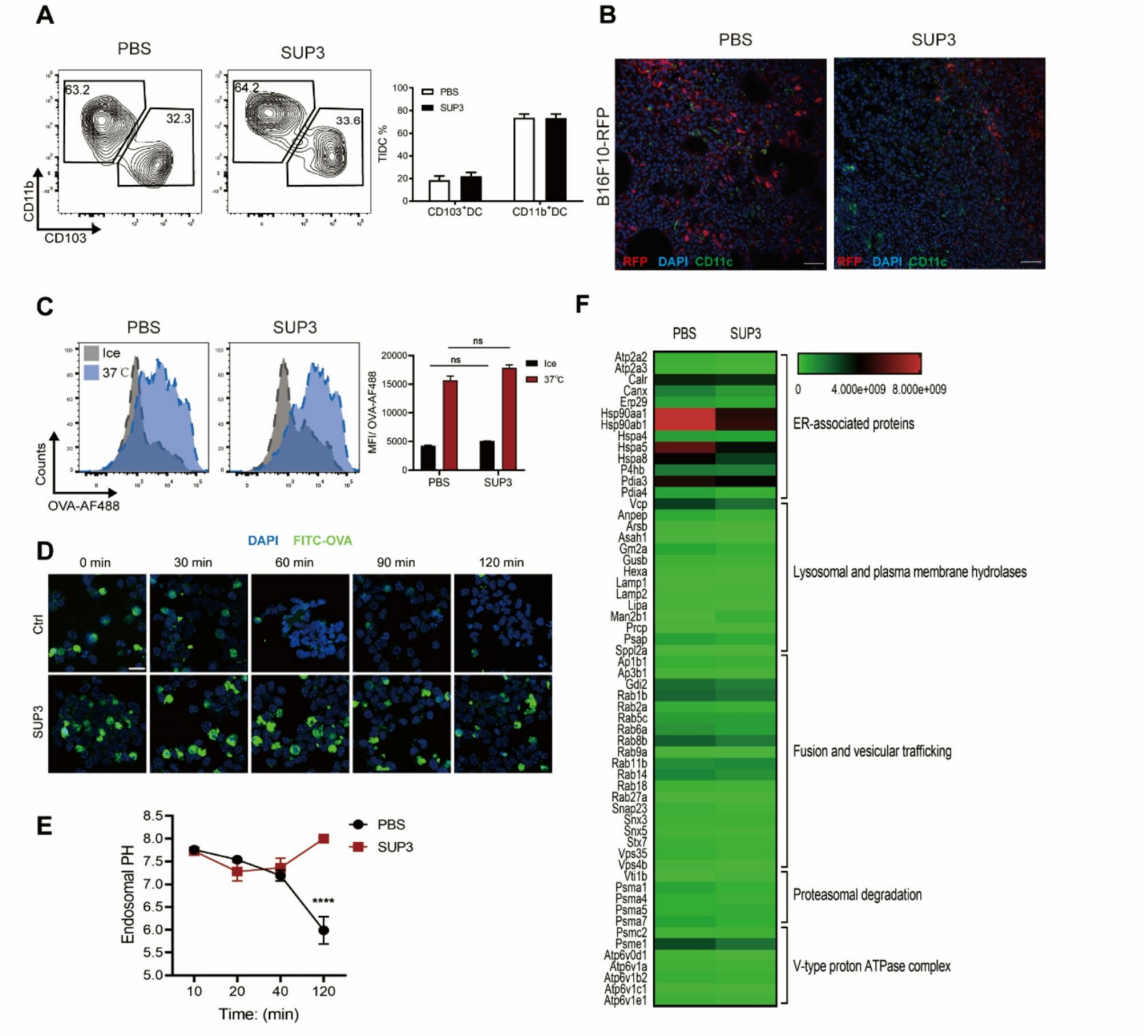
SUP3 promoted antigen deposition in cDC1 in B16F10 or B16F10-RFP tumor-bearing mice.**A**, **B** B16F10 or B16F10-RFP tumor-bearing mice were treated with SUP3 (0.5 mg/kg), and the tumor-infiltrating cDC frequency and the colocalization of tumor cells and DCs were determined. *n* = 3 per group. **C** Flt3L cultured bone marrow-derived cDC1s were pretreated with SUP3 (1 µM) for 4 h and then pulsed with OVA-AF488 (5 µg/mL) for 30 min at 37 °C or on ice as a control. The cells were washed three times with cold PBS, and fluorescence was measured by flow cytometry. **D** cDC1s were pretreated with SUP3 (1 µM) for 4 h and then pulsed with OVA-AF488 (5 µg/mL) for 30 min at 37 °C. Cellular fluorescence intensity was measured at the indicated times (15, 30, 60, and 120 min). **E** cDC1s were pretreated with SUP3 (1 µM) for 4 h and pulsed with FITC-dextran (1 mg/mL) for 15 min at 37 °C. The process was then stopped, and the cells were washed with cold PBS. The cellular MFI was analyzed by flow cytometry at the indicated times. The detected pH values were normalized to a standard curve generated from cells pulsed in solutions with different pH values ranging from 4 to 8. **F** Splenic cDC1s were purified and treated with SUP3 (1 µM) for 24 h. Total protein was extracted and analyzed by LC/MS. **A**, **C**, **E** Data are shown as the mean ± SEM. (A-F) Results are from 2 to 3 independent experiments. Data were analyzed by two-way ANOVA **A**, **C**, **E**. *ns* not statistically significant, ****P < 0.0001. ANOVA, analysis of variance

CD103^+^ cDC1s are the dominant intratumoral APCs transporting tumor antigens to the TdLNs [6]. To investigate the underlying mechanism of SUP3-promoted antigen cross-presentation by cDC1s, B16F10 tumor-bearing mice were treated with SUP3. No significant increase in the frequency of CD103^+^ cDC1s was observed compared to the control group (Fig. 4A). A similar DC infiltration phenomenon was also observed in B16F10-RFP tumors, in which the frequency of CD11c^+^ DCs (green fluorescence) did not increase (Fig. 4B). To analyze the mechanism of enhanced cross-antigen presentation of SUP3 treated cDC1s, we used a Flt3L-suplemented DC culture (FLDC) system to generate cDC1s. We then tested the ability of antigen uptake by SUP3 treated cDC1s. When the cDC1s were pulsed with Alexa Fluor 488 (AF488)-conjugated ovalbumin (OVA), the antigen endocytosis by these cDC1s was not affected by SUP3 treatment (Fig. 4C). However, after cDC1 endocytosed OVA for 15 min, then followed by the chasing of antigen deposition at different time points, we found that SUP3 prolonged the maintenance of antigen in the cells, and delayed the initial digestion of antigen that typically occurs 30 min after OVA pulsing (Fig. 4D). It had been shown that lysosome acidification facilitated rapid antigen degradation [17], we also observed that SUP3 treatment inhibited this lysosome acidification process and the decrease of cellular endosomal pH, then subsequently slowed down the process of antigen degradation compared to that in control cDC1s (Fig. 4E), which was beneficial for antigen cross presentation. Furthermore, liquid chromatography/mass spectrometry (LC/MS) data revealed that SUP3-treated cDC1s exhibited a relatively slower antigen processing state, reflected by the reduced expression of proteins related to antigen processing (Fig. 4F).

Interestingly, SUP3 also promoted a relatively immobile perinuclear lysosome pool near the microtubule-organizing center (MTOC), as shown by electron microscopy, which enhances the antigen cross-presentation by DC (Supplementary Fig S5A). The perinuclear cloud cluster appears to be a site for the efficient maturation of endosomes [18], and it had been shown that lysosome perinuclear clustering could promote DC antigen cross-presentation [19]. We observed a similar phenomenon by confocal microscopic analysis (Supplementary Fig S5B). These findings suggested that SUP3 promoted cDC1 antigen cross-presentation by delaying antigen degradation.

**Fig. 5:**
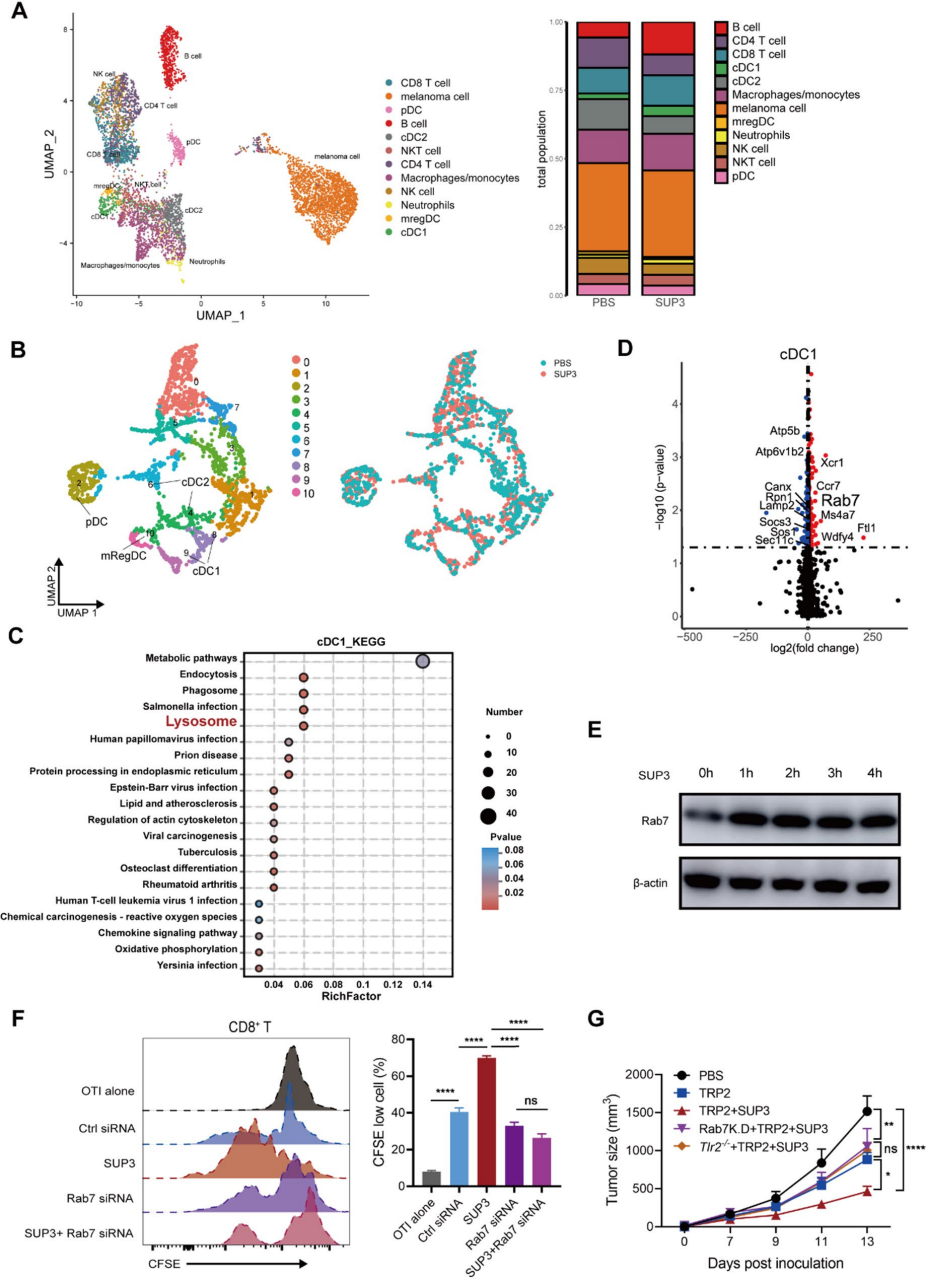
Rab7 induced by SUP3 is required for cDC1 antigen cross-presentation and tumor eradication. **A** Notably, 7 days after SUP3 treatment, surgical excision of tumor tissue in B16F10 tumor-bearing mice and reinjection with tumor cells after 30 day) B16F10 tumor-bearing mice were treated with SUP3 (0.5 mg/kg) or PBS. Tumor-infiltrating immune and non-immune cells were then purified for scRNA-seq. UMAP data was shown. **B** The tumor-infiltrating myeloid cells of B16F10 tumor-bearing mice treated with SUP3 (0.5 mg/kg) or PBS were analyzed by scRNA-seq, and UMAP data are shown. **C** KEGG pathway enrichment analysis of upregulated differentially expressed genes (fold change > 2) in cDC1 cells from the SUP3-treated group compared to the PBS control. **D** Differential gene expression analysis in cDC1s between SUP3 and PBS treated B16F10 tumor-bearing mice. **E** Rab7 expression in cDC1s was measured by Western blotting at various time points (0, 1, 2, 3, and 4 hours) after SUP3 stimulation. **F** FLDC1s (1 × 10^4^) were transfected with Rab7 siRNA, pretreated with SUP3 (1 µM), and/or pulsed with OVA (50 µg/mL) for 4 hours before being mixed with purified 1 × 10^5^ CD8^+^ OT-I T cells for 72 h. T-cell proliferation was detected by FACS. (G) FLDC1s from wild-type mice or *Tlr2*^–/–^ mice were sorted, and wild-type FLDC1s were transfected with Rab7 siRNA or TRP2 (50 µg/mL)-pulsed cDC1s that were pretreated with SUP3 (1 µM) and injected into B16F10 tumor-bearing mice on days 3 and 7 after tumor inoculation. Tumor size was measured; *n* = 6 per group. **F**, **G** Data are shown as the mean ± SEM. **E**-**G** Results from 2 to 3 independent experiments. Data were analyzed by two-way ANOVA (**F**, **G**). *ns* not statistically significant, *P < 0.05, **P < 0.01, ***P < 0.001, ****P < 0.0001

To further unravel the immune microenvironment of SUP3-treated B16F10 melanoma, we collected tumor-infiltrating immune cells and nonimmune cells for transcriptome analysis by single-cell RNA sequencing (scRNA-seq). We identified several immune cell types, including T cells, B cells, NK cells, and myeloid cell subsets, including macrophages, DCs, and granulocytes (Fig. 5A). Notably, the same subtypes of cells were found in both SUP3-treated and saline-treated tumor-bearing mice, no differential clusters that appeared in either groups. Subsequently, we performed unsupervised clustering of myeloid cells and identified a total of 11 clusters consistent with the myeloid lineage, comprising 5 clusters of monocytes/macrophages and 6 clusters of cDCs (Fig. 5B). The tumor-infiltrating DCs also expressed high levels of major histocompatibility complex (MHC)-II molecules. In addition, we found that CLEC9A^+^ cDC1s expressed classical cDC1 marker genes, such as *Xcr1*, *Irf8*, *Batf3*, *CD24a*, and *Itgae*, whereas cDC2s expressed *S100b*, *Irf4*, and *Fcer2a* (Supplementary Fig S5C). Furthermore, pDCs showed high *Siglech* expression. Interestingly, a cluster of mature DCs enriched in immunoregulatory molecules (mRegDCs, LAMP3^+^) expressed *Batf3*, indicating a possible origin from cDC1s (Supplementary Fig S5C). In accordance with previous LS/MS findings, the protein processing and presentation signal genes of cDC1s were enriched in the SUP3 treatment sample, and the upregulated genes were mainly involved in antigen processing and presentation (Fig. 5C and D). These findings indicated that immune cells, particularly DCs, play a crucial role in restraining tumor progression after SUP3 treatment. Among these upregulated genes, a gene encoding small GTPase family proteins involved in antigen processing, endosome transport and fusion with the lysosome was identified [20, 21]. Combined with previous scRNA-seq data, we found that SUP3 upregulated GTPase Rab7 expression (Fig. 5E), which was known to promote lysosomal movement towards the MTOC and antigen cross-presentation [22, 23]. Knockdown of Rab7 expression by siRNA in cDC1s (Supplementary Fig S5D) led to a decline in antigen cross-presentation after SUP3 treatment, resulting in a low level of antigen-specific T-cell proliferation (Fig. 5F). Moreover, the antitumor effect of SUP3 was eliminated when antigen-pulsed Rab7 knockdown cDC1s were adoptively transferred into the tumor-bearing mice intravenously (Fig. 5G). Our findings suggested that the small GTPase Rab7 was required for SUP3 promoted cDC1 antigen cross-presentation.

### SUP3 induced PD-L1 upregulation on DCs and anti-PD-L1 synergized with SUP3 in anti-tumor response

**Fig. 6:**
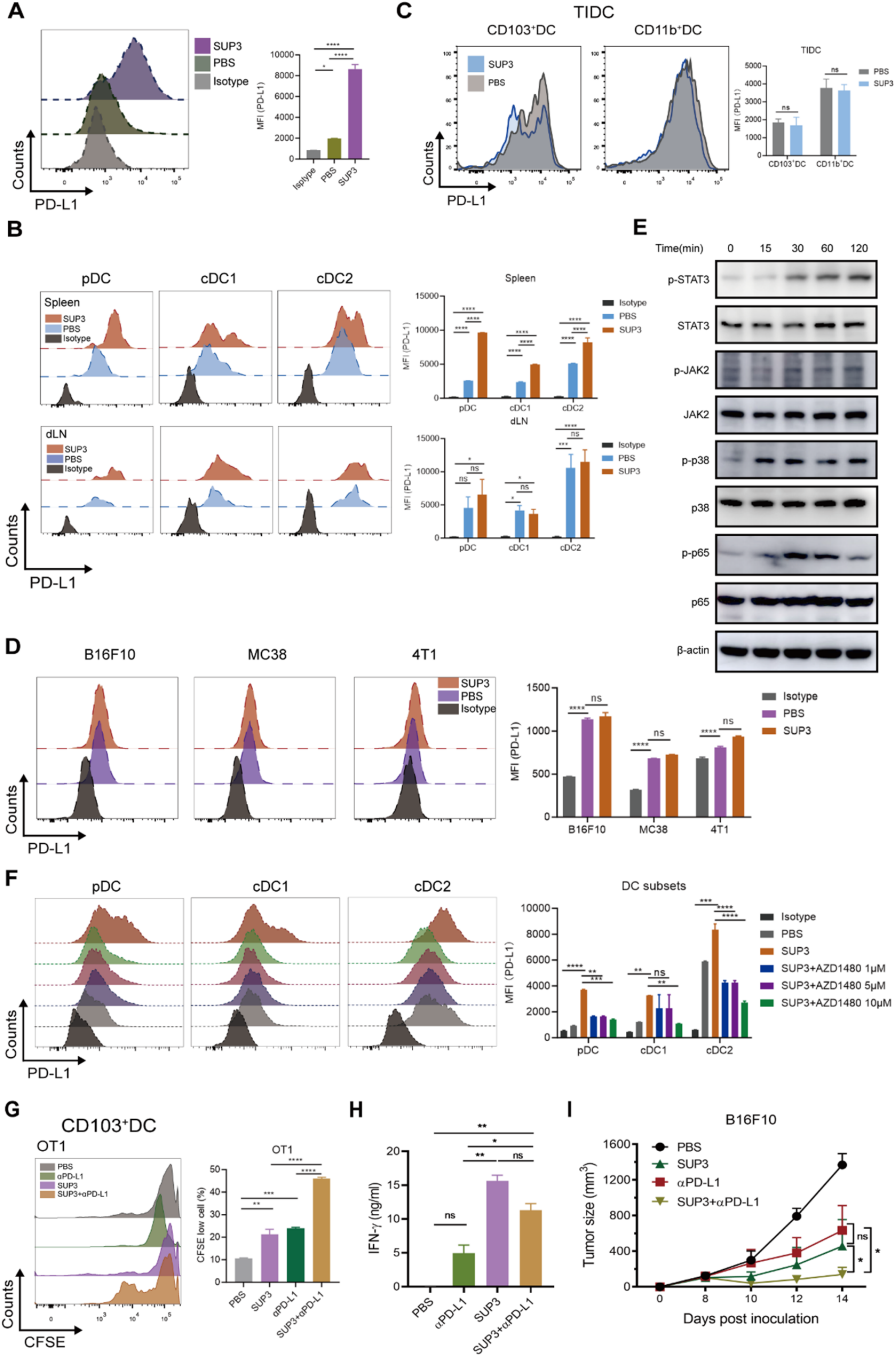
SUP3 combined with anti-PD-L1 Ab treatment exerted synergistic effects on the antitumor response.**A** FLDCs were stimulated with SUP3 (1 µM), and PD-L1 expression was detected 24 h later. **B** Spleen and dLN DC subsets were sorted and stimulated with SUP3 (1 µM) for 24 h, and PD-L1 expression was detected. **C** B16F10 tumor-bearing mice were treated with SUP3 (0.5 mg/kg), and the PD-L1 expression of tumor-infiltrating cDCs was measured. **D** B16F10, MC38 and 4T1 tumor cells were treated with SUP3 (1 µM), and PD-L1 expression was detected 24 h later. **E** cDC1s were stimulated with SUP3 (1 µM), and signaling pathway proteins (JAK2, p-JAK2, STAT3, p-STAT3, p38, p-p38, p65, and p-p65) and β-actin were measured at the indicated times. **F** Flt3L culture derived DC subsets were treated with SUP3 (1 µM) and titrated concentrations of a JAK2 inhibitor (AZD1480) for 24 h, and PD-L1 expression was detected. **G** Tumor-infiltrating CD103^+^ cDC1s were sorted from B16F10-OVA tumor-bearing mice and treated with SUP3 (1 µM) and/or anti-PD-L1 Ab (10 µg/mL). cDC1s were cocultured with OT-I mouse-derived CD8^+^ T cells. T-cell proliferation was detected after 72 h. The secreted IFN-γ was measured (**H**). **I** B16F10 tumor-bearing mice were treated with SUP3 by paratumoral injection (0.5 mg/kg every 2 days) and/or combined with intraperitoneal injection of PDL1 Ab (150 µg/mouse, twice a week) on day 8 post-tumor inoculation, and tumor size was measured; *n* = 4–5. (**H**, **I** Data are shown as the mean ± SEM. (A‒K) Results are from 2 to 3 independent experiments. Data were analyzed by two-way ANOVA (**H**, **I**). *ns* not statistically significant, *P < 0.05, **P < 0.01

Based on previous findings, SUP3 facilitated the maturation of immature DCs. Interestingly, we also found that SUP3 induced mature DCs to express the inhibitory molecule PD-L1 in vitro (Fig. 6A). Moreover, SUP3 induced PD-L1 expression on DCs in spleens and lymph nodes (Fig. 6B). Meanwhile, the tumor-infiltrating CD103^+^ cDC1s and CD11b^+^ cDC2s already expressed relatively higher levels of PD-L1 in B16F10 tumor-bearing mice, which was not further enhanced by SUP3 treatment (Fig. 6C). Although PD-L1 was intrinsically expressed on B16F10 or MC38 tumor cells, SUP3 treatment did not enhance PD-L1 expression, not even on 4T1 tumor cells that were PD-L1-negative (Fig. 6D). It has been suggested that PD-L1 expression involves a distinct pathway between tumor cells and immune cells [24]. Based on previous studies, the cytokines IL-6 and TNF-α might participate in this regulatory process [25]; However, even though SUP3 induced IL-6 and TNF-α expression by DCs (Supplementary Fig S6A), PD-L1 expression was not altered even after IL-6 and TNF-α neutralization (Supplementary Fig S6B). Although it had been proposed that IFN-γ could induce PD-L1 expression [26, 27], the expression of PD-L1 on SUP3-treated DCs was not affected when blocked with antibody against IFN-γ (Supplementary Fig S6C). In addition, SUP3 treatment did not induce IFN-γ expression by DCs (Supplementary Fig S6A). These data therefore suggested that SUP3 induced PD-L1 expression by DCs ex vivo through an IFN-γ-independent pathway. Most importantly, we found that almost all ligands of TLR families (R848, PolyI: C, CpG, LPS, Pam2SCK4) or protein (OVA) that activate DC maturation could lead to the expression of PD-L1 (Supplementary Fig S6D). These data suggested that SUP3 facilitated the maturation of immature DCs and promoted the expression of PD-L1 by mature DCs.

It appears that the high levels of PD-L1 expression on tumor-infiltrating DCs could be the results of tumor microenvironment that modulated the expression of PD-L1 on DCs. To understand how the expression of PD-L1 was regulated, we analyzed the relevant signaling pathways, and found that SUP3 induced Jak2/Stat3 phosphorylation and p38/NF-κB signaling in DCs (Fig. 6E). To validate the function of Jak2/Stat3 signaling, a Jak2/Stat3-specific inhibitor, AZD1480 was used. We found that PD-L1 expression on Flt3L cultured DCs was inhibited by AZD1480 even in the presence of SUP3 in vitro (Fig. 6F). Furthermore, through ChIP-qPCR analysis, we found that SUP3 promotes NF-κB p65 binding to the promoter region of PD-L1 (Supplementary Fig S6E).

It had been noted that PD-L1 expression on DCs influenced the efficacy of PD-L1 blockade immunotherapy [28, 29]. To clarify the role of PD-L1 on tumor-infiltrating cDC1s, purified CD103^+^ cDC1s from tumor tissue were treated with SUP3 or PD-L1 Ab individually or in combination. We found that a combination of SUP3 and PD-L1 blockade exhibited synergized effects on antigen-specific T-cell proliferation and IFN-γ expression in vitro (Fig. 6G and H). Furthermore, we observed that the combination of SUP3 and PD-L1 blockade had a greater effect on suppressing tumor growth than either treatment alone (Fig. 6I). Interestingly, in the PD-L1-negative 4T1 tumor model, SUP3 exhibited a better antitumor effect than PD-L1 blockade alone (Supplementary Fig S6F). These results demonstrated that PD-L1 blockade could enhance the efficacy of SUP3-mediated antitumor immunity.

### The antitumor immunity induced by SUP3 could be further enhanced by Flt3L treatment and PD-L1 blockade

**Fig. 7:**
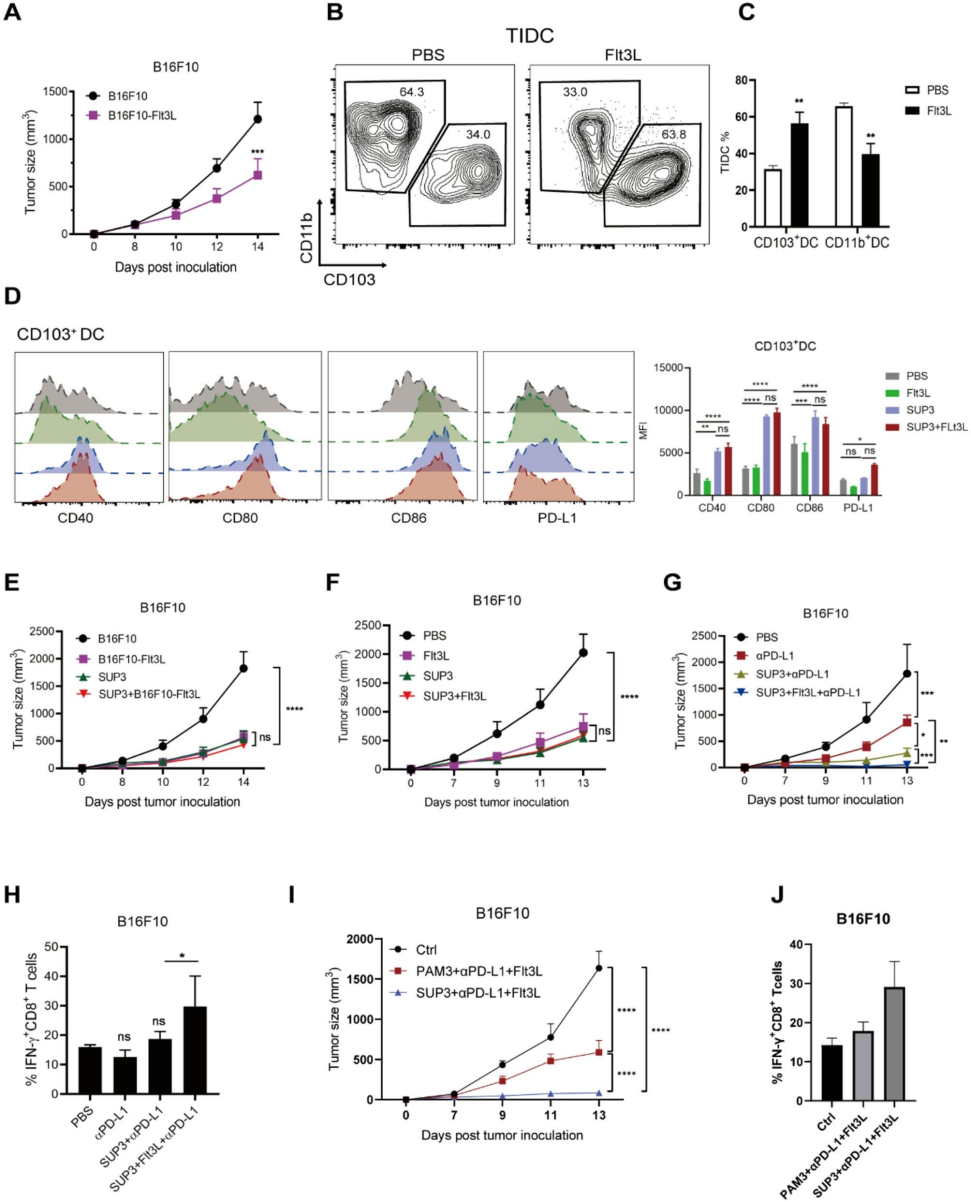
SUP3 and Flt3L synergize with PD-L1 blockade to enhance the antitumor response.**A** C57BL/6 wild-type mice were inoculated with B16F10 or B16F10-Flt3L tumor cells, and the tumor size was determined; *n* = 6 per group. **B**, **C** B16F10 tumor-bearing mice were administered 30 µg Flt3L or PBS for nine consecutive days, and the infiltration of cDC subsets was assessed; *n* = 4. **D** B16F10 tumor-bearing mice were treated with Flt3L (30 µg/mouse) and/or SUP3 (0.5 mg/kg), and the expression of costimulatory molecules (CD40, CD80, and CD86) and PD-L1 in CD103^+^ cDC1 were measured; *n* = 5. **E** C57BL/6 wild-type mice were inoculated with B16F10 or B16F10-Flt3L tumor cells and treated with SUP3 (0.5 mg/kg), and tumor size was determined; *n* = 5. **F** B16F10 tumor-bearing mice were treated with Flt3L (30 µg) and/or SUP3 (0.5 mg/kg), and the tumor size was determined; *n* = 5. **G** B16F10 tumor-bearing mice were treated with SUP3 (0.5 mg/kg/every 2 days), Flt3L (30 µg/mouse) and/or Flt3L combined with PDL1 Ab (150 µg/mouse, twice a week), and tumor size was measured; *n* = 5. Additionally, the IFN-γ secreted by tumor-infiltrating CD8^+^ T cells was detected **H**. **I** B16F10 tumor-bearing mice were treated with SUP3 or PAM3 (0.5 mg/kg/every 2 days), Flt3L (30 µg/mouse) combined with PDL1 Ab (150 µg/mouse, twice a week), and tumor size was measured; *n* = 5. **J** The IFN-γ secreted by tumor-infiltrating CD8^+^ T cells was detected. **A**-**H** Results are from 2 independent experiments. Data are shown as the mean ± SEM. Data were analyzed by one-way ANOVA **C**, **D**, **H**, **J** and two-way ANOVA **A**, **E**, **F**, **G**, **I**. *ns* not statistically significant, *P < 0.05, **P < 0.01, ***P < 0.001, ****P < 0.0001

The tumor-infiltrating DCs, particularly CD103^+^ cDC1s, were essential for eliciting CD8^+^ T cell responses [9], and the abundance cDC1 in tumors could be used to predict responsiveness to anti-PD-1 blockade [30]. To test whether increasing the number of tumor-infiltrating DCs could further enhance the antitumor effect of SUP3 and PD-L1 blockade combination therapy, we used a B16F10-Flt3L tumor model, in which the B16F10 tumor cells could produce the hematopoietic growth factor Flt3L. Flt3L is an essential cytokine for DC differentiation, we found that in the presence of Flt3L tumor growth was inhibited (Fig. 7A). Then, to provide a more accurate measurement, we injected the B16F10 tumor-bearing mice with Flt3L for nine consecutive days and found that Flt3L administration increased the frequency of tumor-infiltrating CD103^+^ cDC1 in vivo, which represented the dominant myeloid cell population within the tumor (Fig. 7B and C). Moreover, following treatment with the combination of SUP3 and Flt3L, CD103 + cDC1s within the tumor continued to exhibit high expression of co-stimulatory molecules, such as CD40, CD80, and CD86 expression (Fig. 7D). However, SUP3 combined with Flt3L treatment did not demonstrate a synergistic antitumor effect in comparison to SUP3 or Flt3L treatment alone, either in the B16F10-Flt3L tumor model or after systemic administration of Flt3L (Fig. 7E and F). In line with the ex vivo studies, SUP3 administration significantly accelerated the maturation of immature CD103^+^ cDC1s and increased the expression of PD-L1 (Fig. 6A), the above results suggested that PD-L1 upregulation on DCs might limit the therapeutic efficacy of the combined treatment of SUP3 and Flt3L. Thus, we further investigated the outcomes of a triple combination of SUP3, Flt3L and PD-L1 blockade (Supplementary Fig S7A), and observed that this triple treatment exhibited the strongest suppression of B16F10 tumor growth (Fig. 7G). As a result, triple therapy also increased the IFNγ^+^ CD8^+^ T-cell frequency in the B16F10 tumor model (Fig. 7H). Similar results were seen in the MC38 colon cancer model (Supplementary Fig S7B). Furthermore, the triple treatment increased DC infiltration in the MMTV-PyMT breast tumor model, leading to increased CD8^+^ T-cell infiltration and subsequent reductions of CD31^+^ and Ki67^+^ cells compared to the anti-PD-L1 Ab alone group or the SUP3 and anti-PD-L1 Ab combination group (Supplementary Fig S7C and D). Additionally, triple combination therapy dramatically reduced the metastasis of malignant breast tumor cells into the lung tissue (Supplementary Fig S8).

Building on this triple-combination model, we further compared SUP3 with PAM3 (Supplementary Fig S9A). We observed that the “SUP3 + anti-PD-L1 + Flt3L” regimen exhibited stronger suppression of B16F10 tumor growth than the “PAM3 + anti-PD-L1 + Flt3L” combination (Fig. 7I and Supplementary Fig S9B). Concurrently, the “SUP3 + anti-PD-L1 + Flt3L” treatment also increased the frequency of IFN-γ⁺ CD8⁺ T cells in the B16F10 tumor model compared to the PAM3-based triple combination (Fig. 7J and Supplementary Fig S9C). These findings collectively demonstrated that SUP3 and Flt3L work in concert with anti-PD-L1 inhibition to elicit a very effective antitumor immunity.

## Discussion

**Fig. 8:**
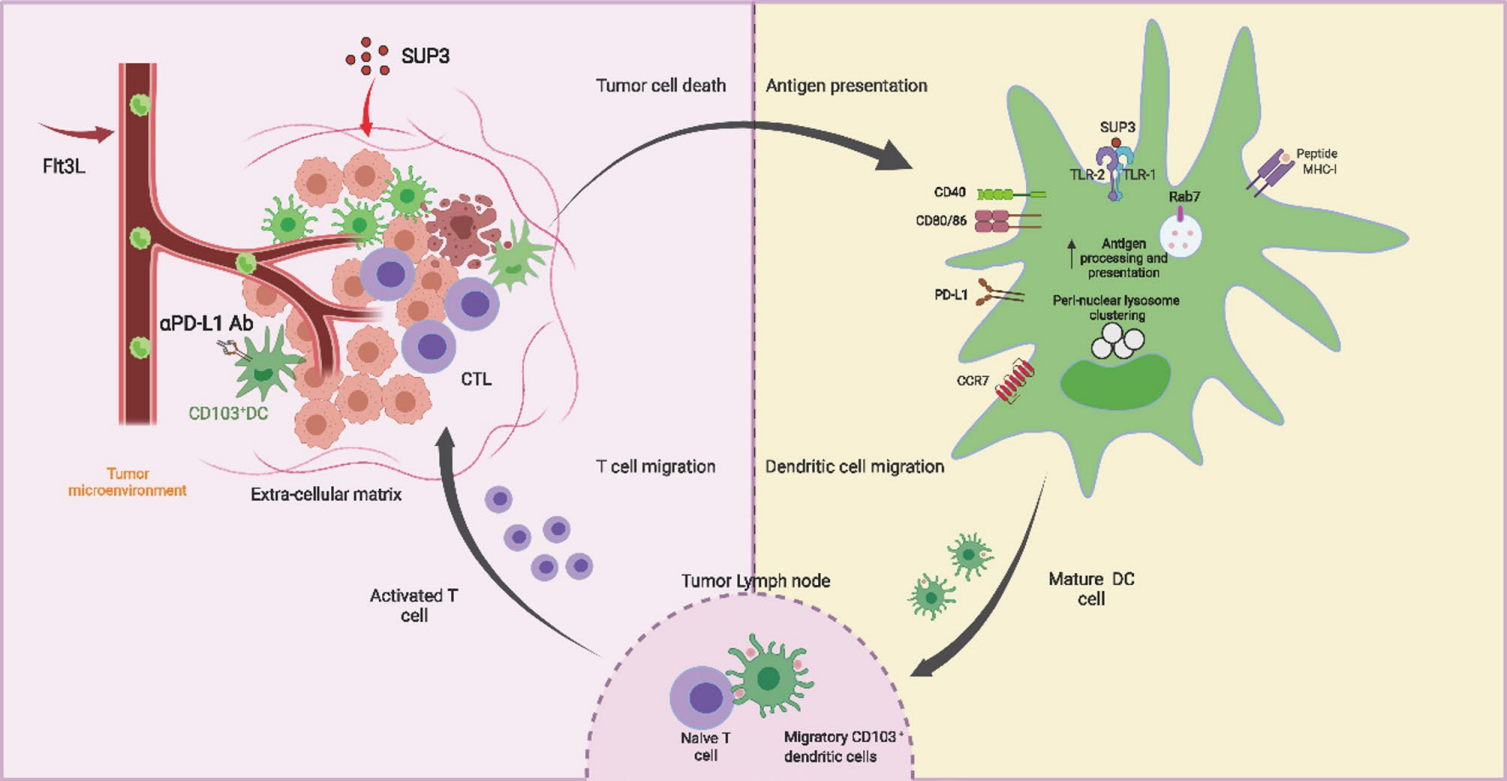
The schematic diagram illustrates the mechanism of SUP3. SUP3 is capable of promoting the maturation of cDC1, enhancing their cross-presentation of antigens, and facilitating their migration to peripheral lymphoid tissues. However, SUP3 can also induce the expression of PD-L1 on dendritic cells (DCs). When PD-L1 is blocked, SUP3 can be used in combination with anti-PD-L1 antibodies to exert synergistic anti-tumor effects. Flt3L can induce cDC1 infiltration into tumors but also leads to the expression of PD-L1. Anti-PD-L1 antibodies can facilitate the combination of SUP3 and Flt3L, thereby inducing the migration of antigen-specific T cells into tumor tissues and enhancing their cytotoxic activity against tumor cells, further augmenting the anti-tumor response

Our previous research demonstrated that the novel TLR2 agonist SUP3 serves as a more effective adjuvant and immune modulator than Pam3CSK4 [15]. However, the molecular mechanisms underlying SUP3’s function and its potential role in antitumor immunity remained incompletely understood. In this study, we revealed that SUP3 enhances antitumor immunity by promoting cDC1 maturation and antigen cross-presentation within the tumor microenvironment more effectively than Pam3CSK4. When SUP3 was combined with PD-L1 blockade and Flt3L administration, a robust antitumor response was achieved through enhanced expansion, maturation, and antigen presentation by cDC1s, leading to subsequent activation of CD8⁺ effector T cells (Fig. 8).

The role of TLR2 signaling in antitumor immunity appears context-dependent and complex. Structural differences among TLR2 agonists, as well as the specific target cell types, may significantly influence functional outcomes [31]. For example, TLR2 activation in nonimmune cells—such as tumor cells or endothelial cells—by endogenous ligands can promote tumorigenesis [32, 33]. Similarly, tumor-derived factors can impair myeloid cell function via TLR2, contributing to an immunosuppressive microenvironment [34, 35]. In contrast, our findings indicate that SUP3-induced TLR2 activation in dendritic cells inhibits tumor growth by driving DC maturation and cross-presentation. Although the precise mechanisms by which TLR2 modulates DC subset functionality, particularly tumor-infiltrating cDC1s, remain incompletely resolved [36, 37], we demonstrated that SUP3 slows endosomal acidification in cDC1s, delaying antigen degradation and thereby facilitating cross-presentation. We also identified SUP3-mediated upregulation of genes associated with DC function, including the small GTPase Rab7, which contributed to enhanced cross-presentation (Fig. 8). Further investigation is needed to explore the roles of other SUP3-upregulated molecules. Tumor-infiltrating DCs often exhibit impaired maturation due to the immunosuppressive tumor microenvironment [38–40]. Therefore, combining SUP3 that induced DC maturation and upregulated PD-L1 expression, with immune checkpoint inhibition could promote a stronger antitumor response by restoring DC activity [41]. Block PD-L1 expressed on DCs synergized with SUP3 treatment, leading to significantly enhanced antitumor immunity, when compared with individual treatment. These findings were in line with those reported by previous studies. Clinical evidence indicates that peripheral DC counts decline during tumor progression, and that higher densities of infiltrating cDC1s and elevated Flt3L levels correlate with improved prognosis [42]. However, Flt3L-expanded DCs frequently remain immature and require additional activation signals. In our study, SUP3 effectively matured these DCs, while subsequent anti–PD-L1 treatment counteracted SUP3-induced PD-L1 upregulation. Thus, the triple combination of SUP3, Flt3L, and anti–PD-L1 generated the most potent antitumor response by synergistically enhancing DC number, function, and immune checkpoint accessibility.

While this study highlights the potential of SUP3 as a superior TLR2 agonist, several limitations should be acknowledged. First, direct comparative studies between SUP3 and Pam3CSK4 under identical therapeutic regimens are limited; future work should include parallel in vivo dose-response and time-course analyses to rigorously quantify their differential effects on DC activation and tumor control. Second, while the association between Rab7 upregulation and enhanced cross-presentation is compelling, the underlying molecular mechanisms require further elucidation. Key questions remain unresolved: for instance, how does SUP3 induce Rab7 protein expression? It is unclear whether SUP3 directly regulates Rab7 transcription or acts through indirect pathways. Future studies employing techniques such as CUT&Tag or ChIP-seq could help identify the direct transcriptional targets of SUP3 signaling. Additionally, the functional relationship between Rab7-mediated vesicle trafficking to the microtubule-organizing center (MTOC), antigen processing, and subsequent loading onto MHC-I molecules warrants further investigation.

In summary, our findings establish that SUP3 enhances antitumor immunity by modulating DC activation and cross-presentation. Its superior efficacy over Pam3CSK4, combined with a favorable safety profile and synergy with existing immunotherapies, positions SUP3 as a promising candidate for clinical translation. Combining DC-targeted agents with immune checkpoint blockade may represent a viable strategy to overcome immunosuppressive networks and improve outcomes in cancer immunotherapy.

## Supplementary Information

Supplementary Material 1.

## Data Availability

All data generated and analyzed in this study have been included within this article and its additional files. Further details are available from the corresponding author upon request.
