# Supplementary material for: Enhancing anti-tumour immunity through modulating dendritic cell activation by combination therapy with a novel TLR2 agonist and PD-L1 Blockade
Source: J Exp Clin Cancer Res. 2025 Nov 25;44:311. doi: 10.1186/s13046-025-03571-9 (PMC12649022; doi:10.1186/s13046-025-03571-9)
Supplement: Supplementary file 1 — Supplementary Material 1. [file 13046_2025_3571_MOESM1_ESM.docx]

**Supplementary Materia
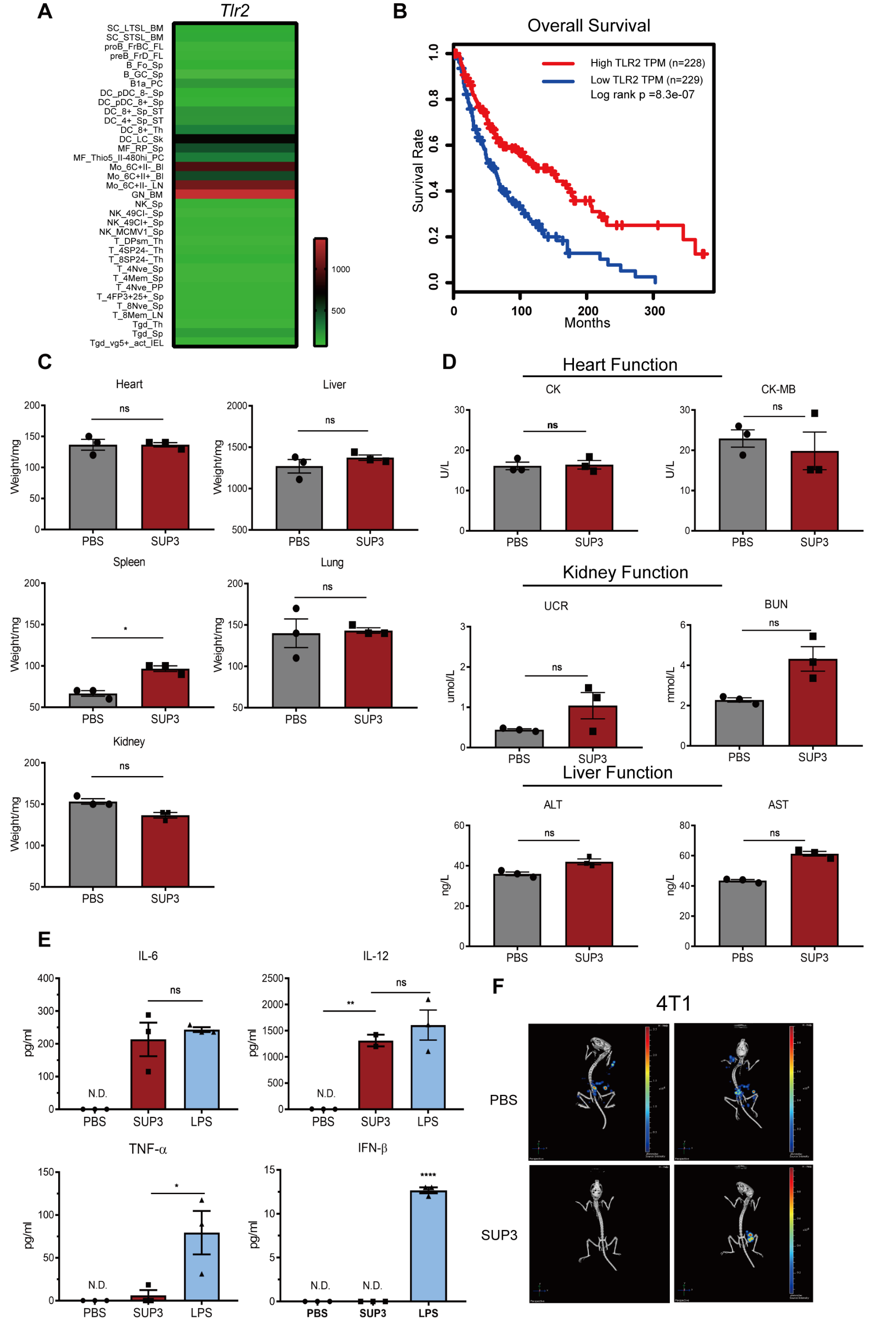
**

**Figure S1. SUP3 treatment did not affect the functions of various organs and induced modest inflammatory cytokine release**.

(A) *Tlr2* expression was analyzed with the Immunogen microarray database. (B) The overall survival of skin cutaneous melanoma patients from the GEPIA database is shown, TPM stands for "transcripts per million". (C, D) Six-week-old male C57BL/6 mice were treated with SUP3 (0.5 mg/kg, subcutaneous injection, every two days, 4 times), and the weights of mouse visceral organs were measured. Serum was also collected, and biochemical indexes were detected; n = 3 for each group. (E) Six-week-old male C57BL/6 mice were treated with SUP3 (0.5 mg/kg, i.v. injection) or LPS (5 mg/kg, i.p. injection), mouse serum was obtained 1 or 4 hours later, and IL-6, IL-12, IFN-β and TNF-α in the mouse serum were detected by ELISA. N.D.: Not detected; n=3. (F) Six-week-old female BALB/c mice were inoculated with 4T1 tumor cells (1×10^6^), and tumor-bearing mice were treated with SUP3 (0.5 mg/kg) or PBS on day 7. Tumor cell metastasis was detected by micro-CT 4 weeks later; n=5. The results are shown as the mean ± SEM from at least two independent experiments analyzed by the log-rank (Mantel‒Cox) test, one-way ANOVA or Student’s unpaired *t* test. ns, not statistically significant; * p<0.05, ** p<0.01.


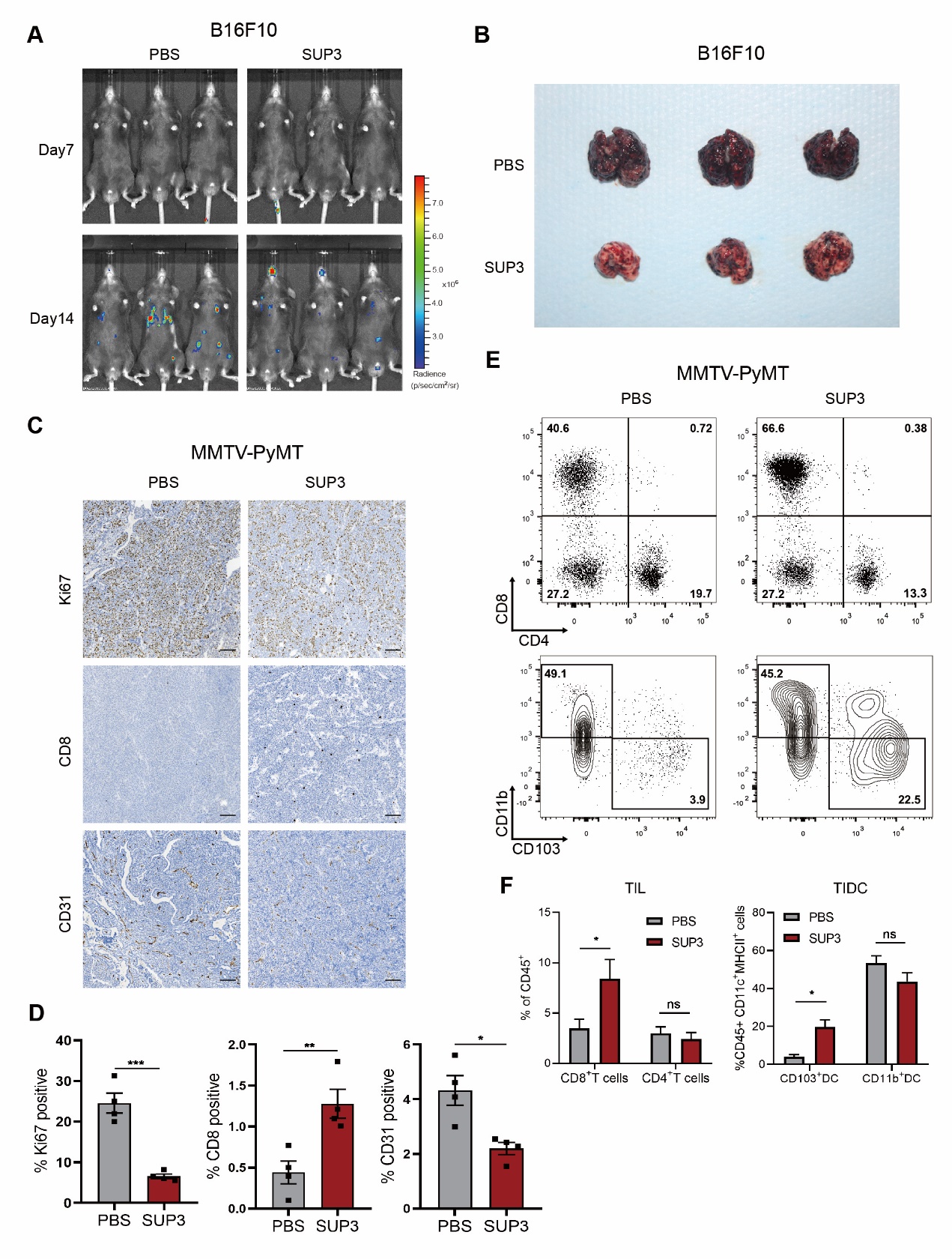


**Figure S2.** **SUP3 inhibited cancer cell growth and metastasis and promoted the recruitment of CD8^+^ T cells and** **cDC1s into tumor tissues.**

(A, B) B16F10 tumor cells (1×10^6^) were injected (i.v.) into C57BL/6 male mice, and tumor-bearing mice were treated with SUP3 (1 mg/kg, intraperitoneal injection, every 2 days) or PBS on day 7. Tumor lesion formation was detected with an IVIS at the indicated time, lung tissue was collected, and pulmonary metastasis was captured on day 15; n=5 for each group. (C, D) MMTV-PyMT mice were treated with SUP3 (0.5 mg/kg, peritumoral injection, every 2 days, 4 times), tumor tissue was collected, and biochemical indexes (Ki67, CD8, and CD31) were detected by IHC. (E, F) MMTV-PyMT mice were treated with SUP3 (0.5 mg/kg, peritumoral injection, every 2 days, 4 times), tumor tissue was enzyme digested, and tumor-infiltrating T cells and DCs were measured by flow cytometry; n=5. The data are representative of two independent experiments; n=5. The results are shown as the mean ± SEM from at least two independent experiments and were analyzed by two-way ANOVA or Student’s unpaired *t* test. ns, not statistically significant; * p<0.05, ** p<0.01, *** p<0.001.


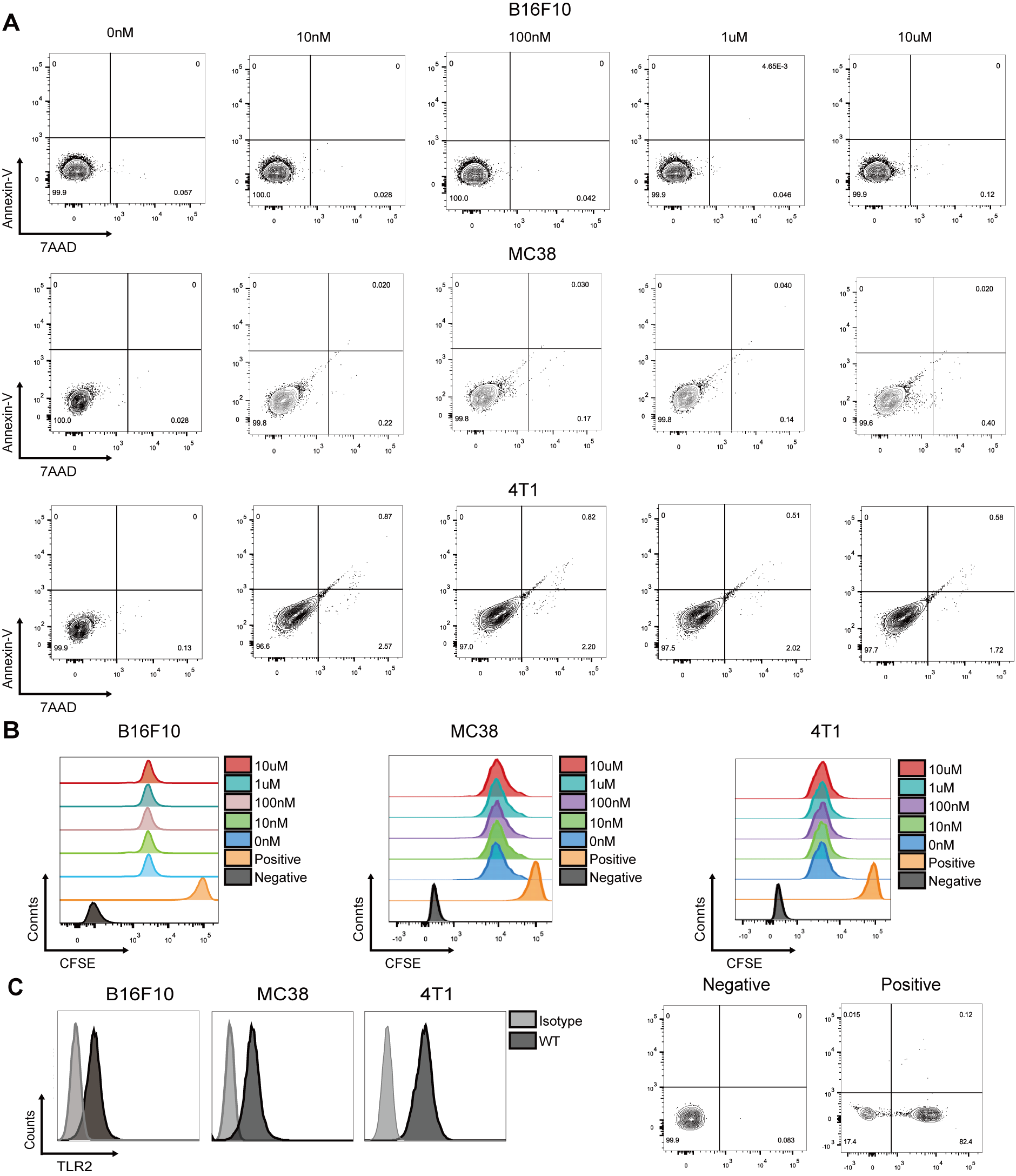


**Figure S3. SUP3 does not affect tumor cell proliferation or apoptosis**

(A, B) Tumor cells were stimulated in vitrowith varying concentrations of SUP3 (0, 10 nM, 100 nM, 1 μM, 10 μM) for 24 hours, followed by flow cytometric analysis of apoptosis (A) and proliferation (B).

(C) Flow cytometry analysis of TLR2 expression on the surface of B16F10, MC38, and 4T1 tumor cells.


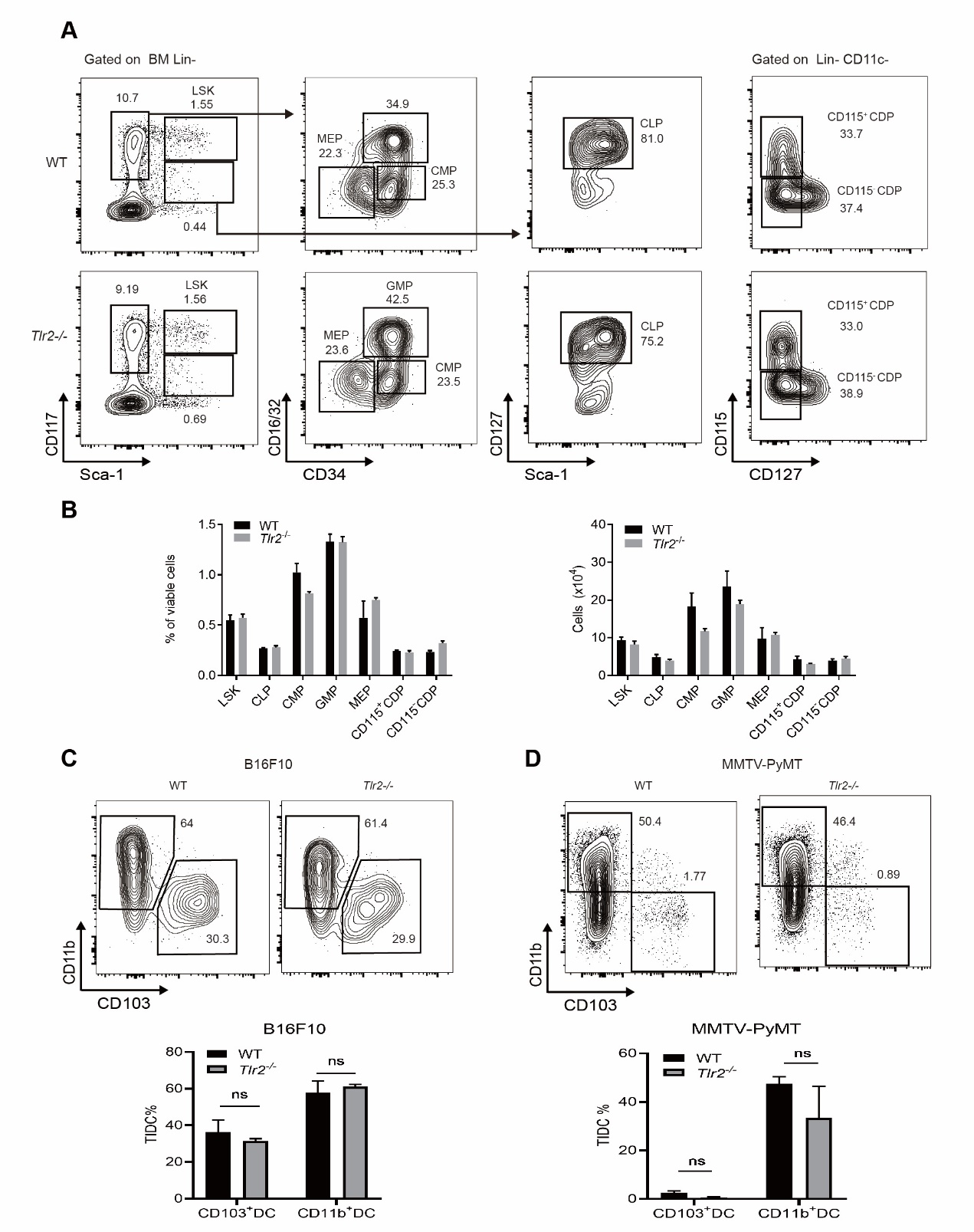


**Figure S4. TLR2 is dispensable for the development and tumor infiltration of DCs**

(A, B) WT and *Tlr2*^–/–^ bone marrow cells were collected and enriched with a Lin antibody cocktail. BM progenitor (LSK, CLP, CMP, GMP, CD115^+^ CDP, CD115^-^ CDP) frequency and numbers were analyzed by flow cytometry; n=3. (C) Six-week-old C57BL/6 wild-type and *Tlr2*^–/–^ mice were inoculated with B16F10 tumor cells (5×10^5^), and tumor-infiltrating cDC subsets were measured; n=3. (D) WT and *Tlr2*^–/–^ MMTV-PyMT mice were obtained, and breast tumor tissue was collected after 12 weeks. Tumor-infiltrating cDC subsets were analyzed by flow cytometry; n=3. The results are shown as the mean ± SEM from two independent experiments and were analyzed by two-way ANOVA. ns, not statistically significant; * p<0.05, ** p<0.01, *** p<0.001.


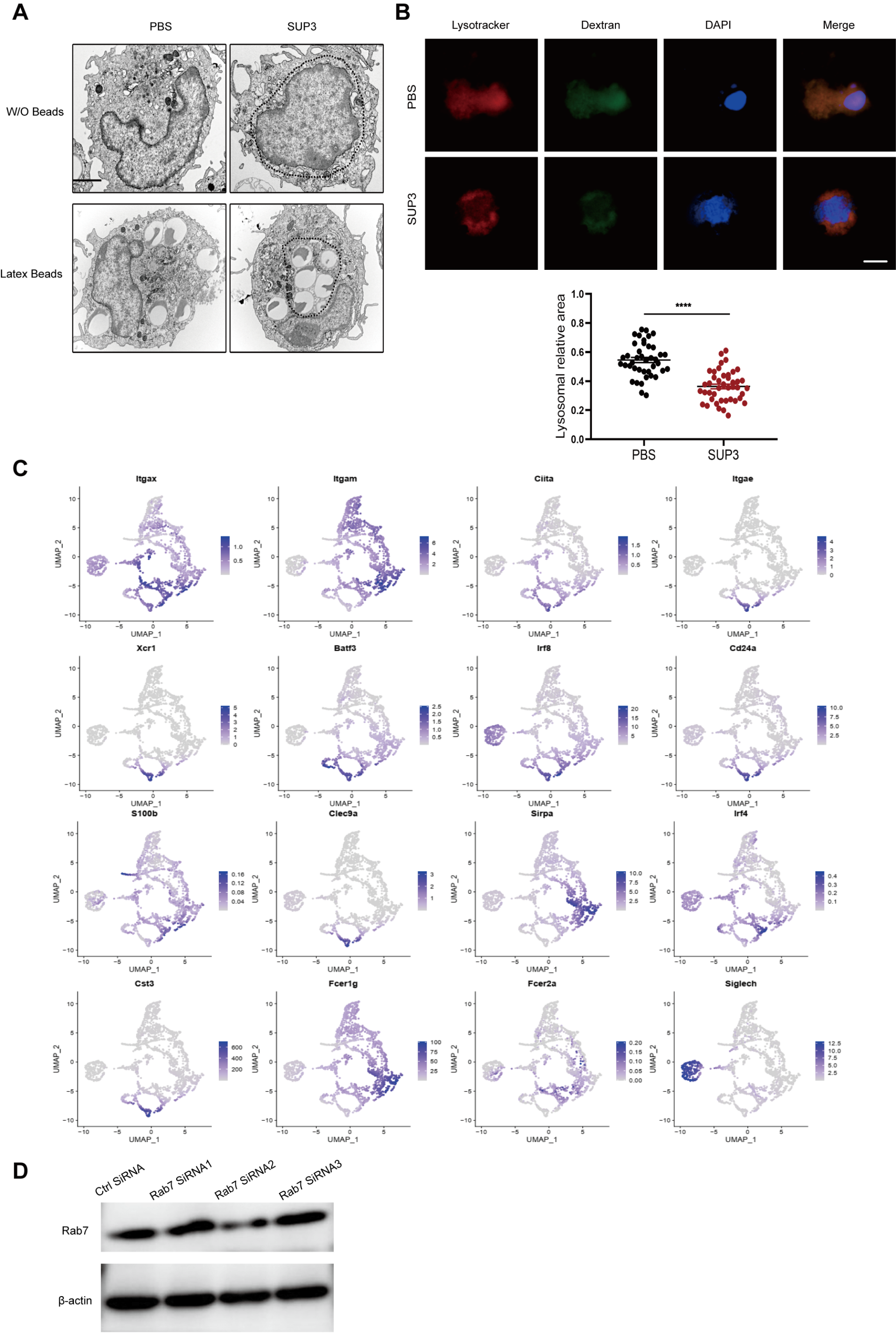


**Figure S5. SUP3 promoted the formation of lysosome perinuclear cloud clusters in cDC1s.**

(A) FLDC1s were sorted and treated with SUP3 (1 µm) for 4 hours or incubated with Latex beads (2 µm) for 15 min. The cells were washed with cold PBS and fixed with 2.5% glutaraldehyde overnight at 4°C. The cells were then dehydrated and permeabilized. Then, they were embedded and sliced. Ultrathin sections were examined by transmission electron microscopy. (B) FLDC1s were sorted and treated with PBS or SUP3 (1 µM) for 4 h, and the cells were pulsed with 100 μg/mL FITC-dextran and LysoTracker Red (1 mM) for 15 min at 37°C. The cells were washed and fixed with 2% PFA. The cells were stained with DAPI, and the fluorescence location was determined with a confocal microscope. (C) B16F10 tumor-bearing mice were treated with SUP3 (0.5 mg/kg) or PBS, and tumor-infiltrating immune and nonimmune cells were sorted for scRNA-seq analysis. (D) FLDC1s were transfected with *Rab7* siRNA, and cellular protein was obtained. Rab7 expression was detected by western blot. Myeloid cell characteristic genes are shown on UMAP. The results are shown as the mean ± SEM from two independent experiments and were analyzed by Student’s unpaired *t* test. ****P<0.0001.

**
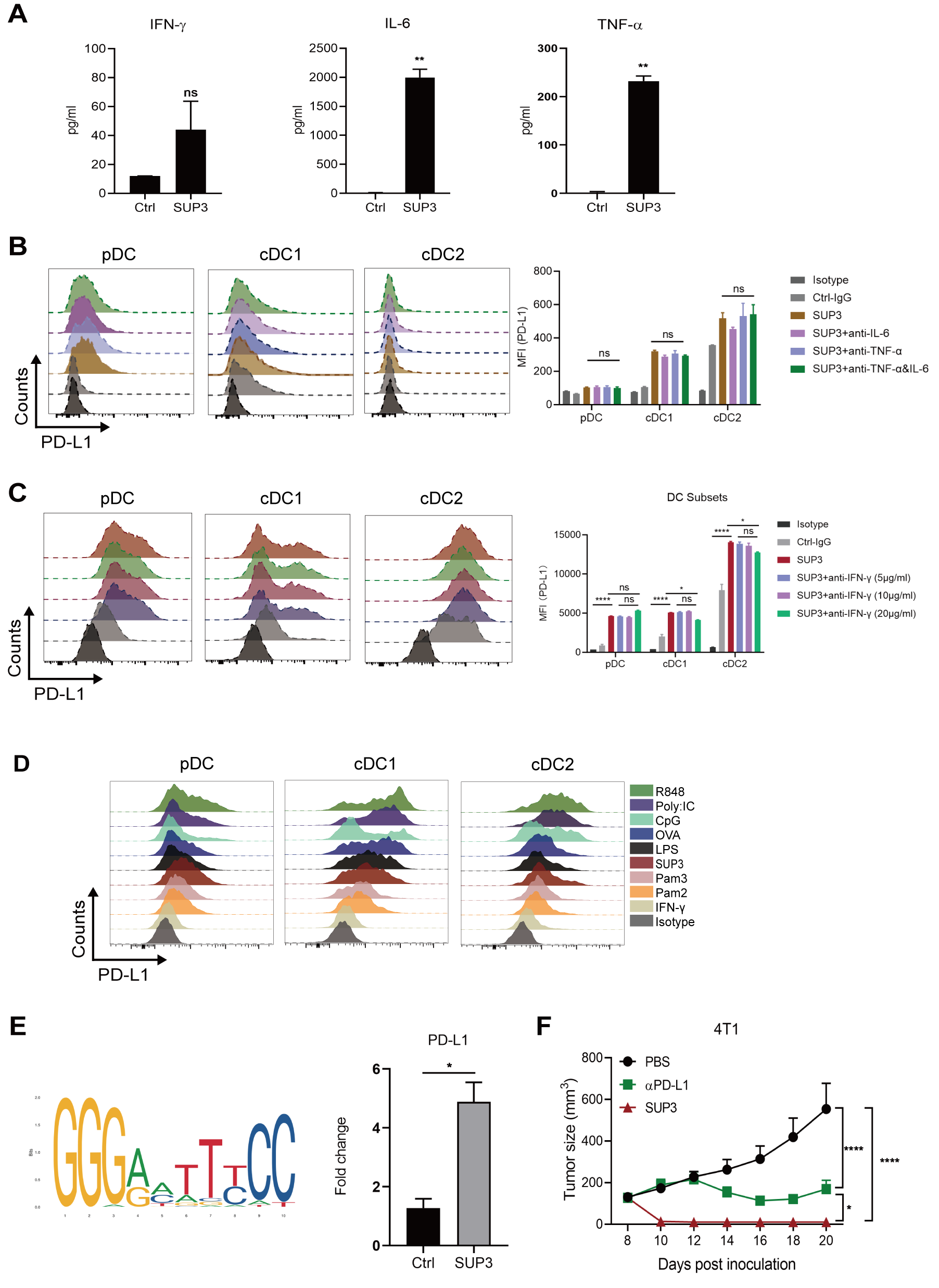
**

**Figure S6. Induction of PD-L1 expression on DCs by SUP3 was IFN-γ independent.** (A) FLDCs were treated with SUP3 (1 µM) for 24 hours, the cell supernatant was collected, and IFN-γ, IL-6 and TNF-α expression was measured by ELISA. (B) Sorted FLDC subsets were treated with SUP3 (1 µM) and titrated concentrations of anti-IL-6 (10 µg/mL) and anti-TNF-α Ab (10 µg/mL) for 24 hours. PD-L1 expression in DCs was detected by flow cytometry. (C) Sorted FLDC subsets were treated with SUP3 (1 µM) and titrated concentrations of anti-IFN-γ Ab (0, 5, 10, and 20 µg/mL) for 24 hours. PD-L1 expression in DCs was detected by flow cytometry. (D) Dendritic cell (DC) subsets generated by Flt3L culture were stimulated with PBS, Pam2CSK4 (50 nM), Pam3CSK4 (1 μM), SUP3 (1 μM), Poly I:C (100 μg/mL), LPS (100 ng/mL), CpG (1 μM), R848 (5 μg/mL), OVA (50 μg/mL), or IFN-γ (40 ng/mL). PD-L1 protein expression was assessed 24 hours post-stimulation. (E)ChIP-qPCR analysis was performed with p65 antibody and PD-L1 promotor-specific primers (binding site: −939 to −930). Data are presented as relative to the resting/PBS group. (F) Six-week-old BALB/c mice were inoculated with 4T1 tumor cells (1×10^6^), and tumor-bearing mice were treated with SUP3 (0.5 mg/kg, paratumoral injection) or anti-PD-L1 Ab (150 µg/mouse, 2 times a week). Tumor size was measured; n=5. The results are shown as the mean ± SEM from two independent experiments and were analyzed by two-way ANOVA or Student’s unpaired *t* test. ns, not statistically significant; * p<0.05, ** p<0.01, **** p < 0.0001.


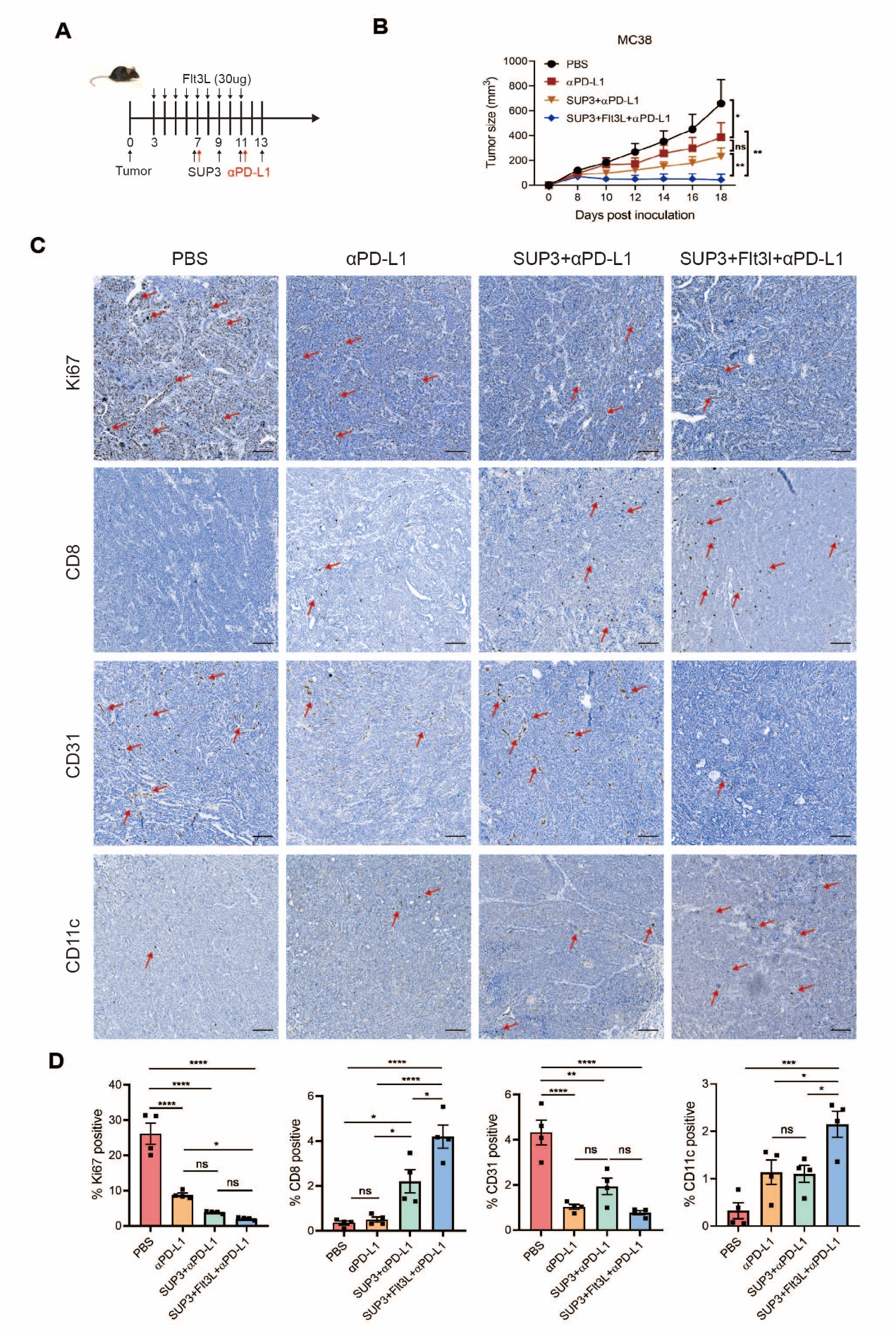


**Figure S7. The antitumor response induced by SUP3 was enhanced by Flt3L and PD-L1 blockade in MC38cc tumor model.**

(A) Drug usage mode. Tumor-bearing mice were treated with SUP3 (0.5 mg/kg, every 2 days), Flt3L (30 µg/mouse for 9 consecutive days from day 3) and/or combined with PDL1 Ab (150 µg/mouse, twice a week) by intraperitoneal injection. (B) MC38 tumor-bearing mice were treated with SUP3 (0.5 mg/kg, every 2 days), Flt3L (30 µg/mouse for 9 consecutive days from day 3) and/or combined with PDL1 Ab (150 µg/mouse, twice a week), and tumor size was measured. The tumor size was determined; n=4-5. (C) MMTV-PyMT mice were treated with SUP3 (0.5 mg/kg, paratumoral injection, every 2 days, 4 times), Flt3L (30 µg/mouse) and/or PDL1 Ab (150 µg/mouse, twice a week). Tumor tissue was collected by intraperitoneal injection, and biochemical indexes (Ki67, CD8, CD31, CD11c) were detected by IHC; n=3. The results are shown as the mean ± SEM from two independent experiments and were analyzed by two-way ANOVA. ns, not statistically significant; * p<0.05, ** p<0.01, *** p<0.001, **** p < 0.0001.


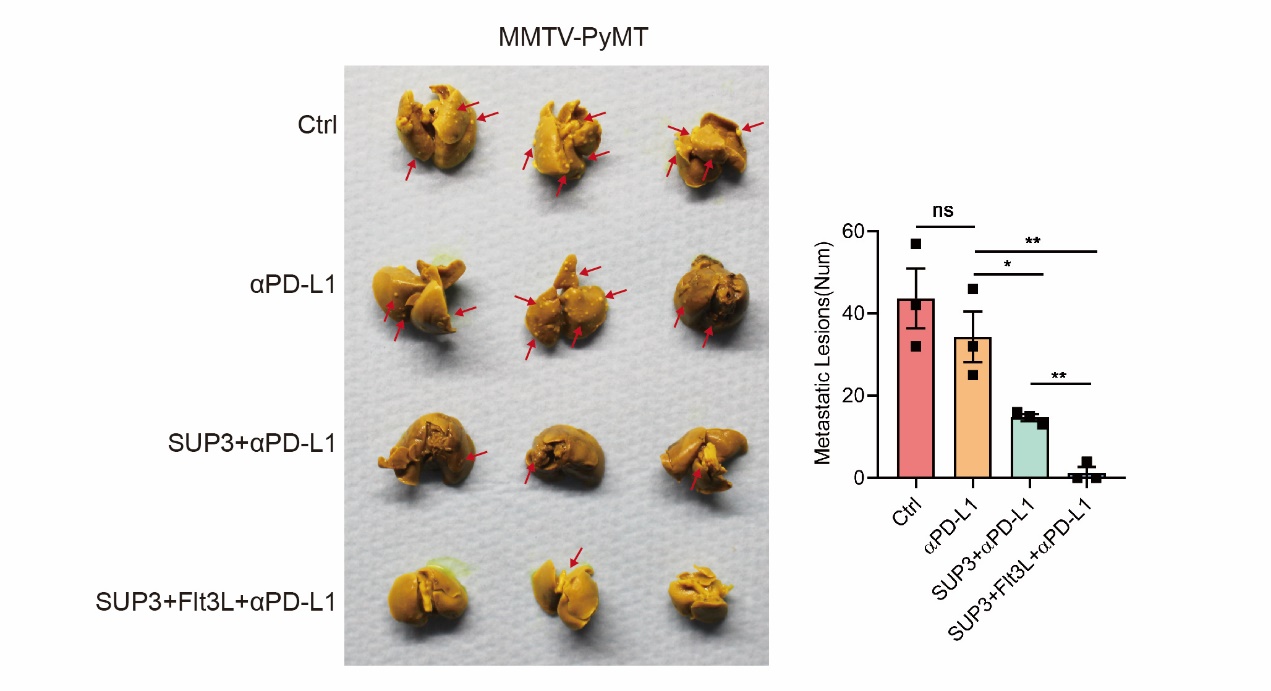


**Figure S8. SUP3 and Flt3L synergize with PD-L1 blockade to inhibit tumor cell metastasis.**

MMTV-PyMT mice were treated with SUP3 (0.5 mg/kg, paratumoral injection, every 2 days, 4 times), Flt3L (30 µg/mouse) and/or Flt3L combined with PDL1 Ab (150 µg/mouse, twice a week) by intraperitoneal injection at 12 weeks. Mouse lung tissue was collected 4 weeks later, and tumor metastatic lesions were measured; n=3 for each group. The data are representative of two independent experiments. ns, not statistically significant. * p < 0.05; ** p < 0.01.


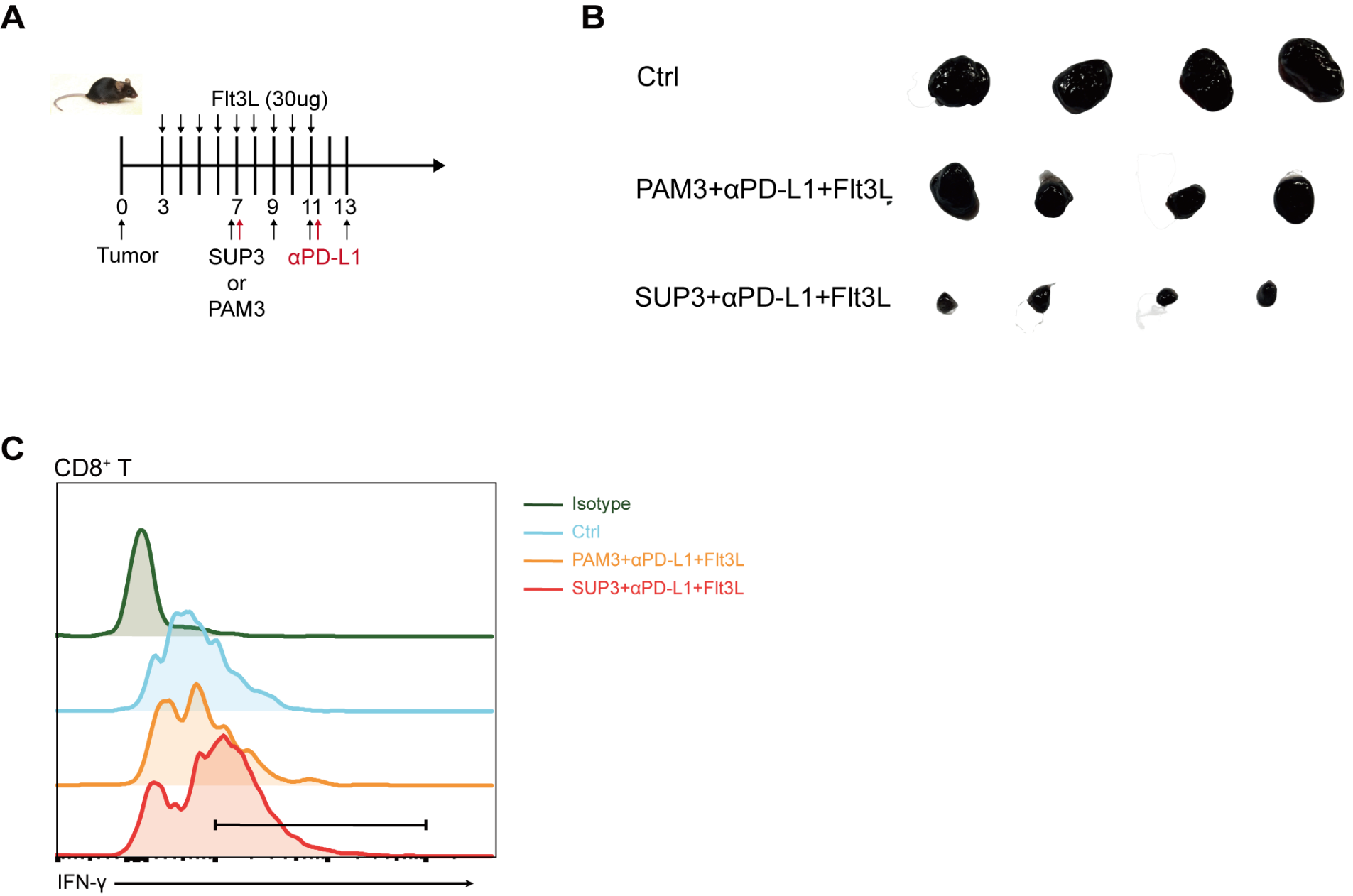


**Figure S9.** The triple combination with SUP3 exhibited significantly stronger anti-tumor response than the triple combination with PAM3

(A-C) B16F10 tumor-bearing mice were treated with SUP3 or PAM3 (0.5 mg/kg/every 2 days), Flt3L (30 µg/mouse) combined with PDL1 Ab (150 µg/mouse, twice a week), and tumor size was measured; n=5.

(D,E) and the IFN-γ secreted by tumor-infiltrating CD8+ T cells was detected (H). (B-E) Data are shown as the mean±SEM. Data were analyzed by one-way ANOVA (E) and two-way ANOVA (B). ns, not statistically significant; *P<0.05, **P<0.01, ***P<0.001, ****P<0.0001.

**Supplementary Methods**

**Mice**

WT C57BL/6 mice (CD45.2^+^), BALB/c mice and BALB/c nude mice were obtained from Charles River, China. CD45.1^+^ C57BL/6 mice, OT-I transgenic mice (C57BL/6-Tg(TcraTcrb)1100Mjb/J), *Ccr2*^–/–^ mice, *Batf3*^–/–^ mice, *Myd88*^–/–^ mice, and *Irf4*^–/–^ mice were purchased from the Jackson Laboratory. *Tlr2*^–/–^ mice, MMTV-PyMT mice (FVB/N-Tg(MMTV-PyVT)634Mul/J) and Tcrbd^–/–^mice (Tcrb and Tcfd double knock out mice) were generously donated by Drs. Jingren Zhang, Hanqiu Zheng and Xuebing Liao respectively (Tsinghua University, Beijing, China). In addition, *Tlr2*^–/–^-deficient mice on the MMTV-PyMT background were backcrossed with MMTV-PyMT mice for >5 generations. Animals were bred and maintained at Tsinghua University in a specific pathogen-free facility at the Laboratory Animal Resources Center. All procedures involving mouse manipulation and all experimental protocols in this study were approved by the Institutional Animal Care and Use Committees of Tsinghua University.

**Cell lines**

B16F10 is a skin melanoma cell line derived from C57BL/6 mice. B16F10-OVA and B16F10-RFP are B16F10 cells transfected with expression vectors encoding OVA and red fluorescent protein, respectively. B16F10-Flt3L is a B16F10 cell line stably expressing Fms-related tyrosine kinase 3 ligand (Flt3L). MC38 is a colon cancer cell line derived from C57BL/6 mice. 4T1 is a breast cancer cell line derived from BALB/c mice. Cells were cultured in Dulbecco’s modified Eagle’s medium (DMEM) (Gibco) supplemented with 10% heat-inactivated FBS (ExCell) and 1% penicillin/streptomycin (Beyotime) at 37°C and 5% CO_2_.

**Preparation of tumor cell suspensions**

Tumor tissue was obtained and digested with 1 µg/mL collagenase IV (Sigma), 0.1 µg/mL hyaluronidase (Sigma) and 1 µg/mL DNase (Roche) at 37℃ for 40 min. Erythrocytes were lysed with RCRB buffer, and debris was removed by filtration through a 70-µm filter. The cell suspension was obtained for further analysis.

**CD8+ T-cell isolation and adoptive transfer**

Spleen or draining lymph node (dLN) CD8^+^ T cells were enriched with the following CD8 negative selection cocktail: anti-CD11b (M1/70, RRID: AB_469344), anti-F4/80 (F4/80, RRID:AB_11141927), anti-TER119 (TER-119, RRID:AB_396936), anti-Gr1 (RB6-8C5, RRID:AB_469739), anti-MHCII (M5/114, RRID: AB_313321), anti-CD19 (ID3, RRID:AB_11154223), anti-B220 (RA36B2, RRID:AB_394457), and anti-CD4 (GK1.5, RRID: AB_2562607). Purified (10-20×10^6^/mL) CD8^+^ T cells were stained with 5 µM CFSE (eBioscience) for 10 min at 37°C. A total of 1-2 × 10^6^ antigen-specific CD8^+^ T cells were adoptively transferred into tumor-bearing mice after 2 hours of SUP3 treatment on day 7 after tumor inoculation. T-cell proliferation in the TdLNs and tumors was measured by FACS after 72 hours.

**In vivo T-cell function assays**

TdLNs and tumor-infiltrating lymphocytes from tumor-bearing mice were enriched by 1.082 g/cm^3^ density gradient centrifugation and then stimulated with PMA (100 ng/mL, MultiSciences, China) and ionomycin (0.5 mg/mL, MultiSciences, China) at 37°C for 4-6 hours, followed by the addition of brefeldin A (10 mg/mL, eBioscience). The cells were blocked with anti-CD16/32, stained for cell surface markers, fixed, permeabilized, and stained with FITC-labeled anti-mouse IFN-γ (eBioscience) and PE-labeled anti-mouse Foxp3 (eBioscience).

**ELISA**

WT C57BL/6 mice were injected intravenously (i.v.) with SUP3 (0.5 mg/kg) or LPS (5 mg/kg, Sigma), and mouse serum was collected after 1 or 4 hours. IL-6, IL-12, TNF-α and IL-β in mouse serum were measured with a mouse ELISA kit. For the mouse biochemical index, mice were injected with SUP3 (0.5 mg/kg) subcutaneously 3 times every other day, and mouse serum was collected at the indicated time. Mouse CK, CK-MB, BUN, UCR and BUN were measured.

**Transmission electron microscopy**

Flt3L cultured bone marrow-derived cDC1s were sorted and treated with SUP3 (1 µm) for 4 hours or incubated with latex beads (2 µm, Sigma) for 15 min. The cells were washed with cold PBS and fixed with 2.5% glutaraldehyde overnight at 4°C. The cells were washed and fixed in osmic acid for 1 hour and then stained with 1% uranyl acetate. The cells were dehydrated with titrated ethanol and permeated with epoxypropane resin. Then, they were embedded and sliced. Ultrathin sections were examined with a Hitachi 7650B transmission electron microscope.

**Generation of bone marrow chimeric mice**

Six-week-old CD45.1^+^ WT mice were lethally irradiated with two doses of 5.5 Gray at a 2-hour interval. CD45.2^+^ WT or CD45.2^+^ *Tlr2*^–/–^, *Myd88*^–/–^, *Batf3*^–/–^, *Irf4*^–/–^ and *Ccr2*^–/–^ mouse bone marrow cells were collected, and 2×10^6^ cells were adoptively transferred into recipient irradiated mice after 6 hours. The mice were fed drinking water containing the antibiotics polymyxin (Amresco) and neomycin (Sigma) for at least 4 weeks. The mice were inoculated with tumor cells at least 8 weeks post-reconstitution.

**In vitro DC stimulation**

Flt3L cultured bone marrow-derived DC subsets were sorted, and pDCs, cDC1s and cDC2s were stimulated with PBS, SUP3 (1 µM) and/or titrated AZD1480 (1 µM, 5 µM, 10 µM, Selleck) for 24 hours. DC surface markers were measured with FITC-labeled anti-mouse MHC-II (Biolegend, RRID: AB_313321), APC-labeled anti-mouse CD40 (eBioscience, RRID: AB_469386), PECy7-labeled anti-mouse CD86 (eBioscience, RRID: AB_313150), PECy7-labeled anti-mouse CCR7 (Invitrogen, RRID: AB_469652), and BV421-labeled anti-mouse PD-L1 (eBioscience, RRID: AB_10897097).

**Endosomal trafficking**

FLDC1s were sorted and treated with PBS or SUP3 (1 µM) for 4 h, and the cells were pulsed with 100 μg/mL FITC-dextran (Invitrogen) and 1 mM LysoTracker Red (Beyotime) for 15 min at 37°C. The cells were washed and fixed with 2% PFA (Servicebio) in PBS for 10-15 min. The cells were stained with DAPI (Vectorlabs), and fluorescence was measured by confocal microscopy (TCS SP8 gSTED 3X, Leica).

**DC progenitor analysis**

WT or *Tlr2*^–/–^ mouse bone marrow cells were collected, and erythrocytes were lysed with RCRB. Cell debris was removed by passing the cells through a 70-µm strainer, and the cells were then suspended in Nycodenz buffer (1.086 g/cm3). Lower-density cells were obtained by centrifugation at 1700 × g for 10 min at 4°C. The Lin^–^ cells were collected with a lineage-negative cocktail: CD2 (rm2.1), CD3ε (KT3-1.1), CD8α (53-6.7), CD45R (RA36B2), CD11b/Mac-1α (M1/70), erythroid lineage (TER119), and Ly6G (1A8). The Lin^–^ cells were stained with monoclonal antibodies: BV605-labeled anti-mouse CD117 (Biolegend, RRID: AB_2562040), APC-Cy7-labeled anti-mouse Sca-1 (Biolegend, RRID: AB_469669), FITC-labeled anti-mouse CD16/32 (Biolegend, RRID: AB_312805), BV421-labeled anti-mouse CD34 (Biolegend, RRID: AB_2650766), BV421-labeled anti-mouse CD127 (Biolegend, RRID: AB_11218800), and PECy7-labeled anti-mouse CD115 (eBioscience, RRID: AB_466564).

**Tissue immunofluorescence and immunohistochemistry (IHC)**

B16F10 or B16F10-RFP tumor-bearing mice or MMTV-PyMT mice were treated with SUP3, anti-PD-L1 Abs, or Flt3L protein. For immunofluorescence, OTC-embedded tumor tissues were sliced into 10 µm thick sections and fixed with cold acetone for 15 min. Slides were blocked with 3% BSA and then stained with a FITC-labeled anti-mouse CD11c monoclonal antibody (CST) and DAPI. The sections were observed with a TCS SP8 gSTED 3X confocal microscope (Leica). For immunohistochemistry, paraffin-embedded slides were dewaxed with titrated xylene/ethanol, washed after deparaffinization and microwaved in EDTA antigen repair buffer. After treatment with 3% H_2_O_2_ for 25 min to block endogenous peroxidase, the slides were incubated overnight at 4°C with anti-mouse CD11c (CST, RRID: AB_2800282), CD8 (Servicebio), Ki67 (Servicebio), CD31 (Servicebio) and PD-L1 (CST, RRID: AB_2799672) monoclonal antibodies. After washing with PBS, the slides were incubated with goat anti-mouse or anti-rabbit secondary antibody (Servicebio) for 50 min at room temperature. The sections were stained with DBA and evaluated with an automatic digital slide scanning system (Axio Scan. Z1, Zeiss).

**Microcomputed tomography (micro-CT) analysis and IVIS images**

Tumor-bearing mice were placed in a chamber connected to an isoflurane anesthesia machine, and the mice were subjected to micro-CT with the following parameters: 90 kV, 80 µA, and 73 mm FOV (QuantμM GX micro-CT, Perkin Elmer). For IVIS imaging, tumor-bearing mice were treated with D-luciferin (0.4 mg per mouse, Cayman) and placed in a dark room connected to an isoflurane anesthesia machine, and live images were captured with a Caliper IVIS Lumina II (Perkin Elmer). Images were analyzed using Living Image 4.0.

**Protein extraction and Western blot analysis**

cDC1s from Flt3L cultured bone marrow were transfected with *Rab7* siRNA or control siRNA or treated with SUP3 (1 µM). The cells were collected and lysed with RIPA buffer supplemented with a protease inhibitor cocktail (Sigma) and phosphatase inhibitor cocktail (Bimake). Cell supernatants were obtained after incubation on ice for 30 min. Proteins were separated by SDS‒PAGE for Western blotting. The PVDF membrane containing protein was blocked with 5% BSA and incubated overnight at 4°C with an anti-mouse Rab7 antibody (CST) and anti-mouse β-actin antibody (CST). The membrane was incubated with a goat anti-mouse or rabbit secondary antibody (ZSGB-BIO) and developed with Chemiluminescent Substrate (Pierce). Images were captured with an Amersham Imager 600 System (GE). Semiquantification of protein in specific bands was performed, and the target protein was analyzed with ImageJ software (<https://imagej>.nih.gov/ij/).

**Chromatin immunoprecipitation (ChIP) followed by qPCR (ChIP-qPCR)**

ChIP-qPCR was performed by using MAGnityTM Chromatin Immunoprecipitation System (Invitrogen, USA) according to the manufacturer’s protocol. In brief, cDC1 cells were crosslinked and chromatin was extracted and sheared. Samples were immunoprecipitated with anti-p65 antibody (CST). The primer sequences used for ChIP-qPCR. The results were from 2 independent experiments followed by normalization to input signals and showed as mean ± SEM.

**Protein sample preparation and quantitative proteomic analysis**

WT mouse spleens were excised and sectioned into pieces, followed by enzyme digestion in a medium containing 1 mg/mL of collagenase III (Worthington) and 0.1 mg/mL of DNase (Roche) for 30 min at 37°C. The cells were then lysed in RCRB buffer to eliminate erythrocytes. Subsequently, the cell debris was removed by straining the cells through a 70-µm filter, and the cells were suspended in Nycodenz buffer (1.077 g/cm3). Lower-density cells were obtained by centrifugation at 1700 × g for 10 min at 4°C. The dendritic cells (DCs) were enriched by using negative cocktails for plasmacytoid DCs (pDCs) and conventional DCs (cDCs), including CD3 (KT3-1.1), CD90/Thy-1 (T24/31.7), TER119 (erythroid), Ly6G (1A8), and CD19 (1D3). Thereafter, the cDC1s were sorted using an AriaIII-2020 (BD) instrument. The cells were then treated with SUP3 (1 µM) or PBS for 24 hours. The treated cell samples were subjected to SDS-PAGE and the gels were stained with Coomassie blue fast staining solution (Beyotime). The gels were cut into pieces and reduced with dithiothreitol (5 mM) in ammonium bicarbonate buffer (50 mM) for 55 min at 55°C. The samples were dried with acetonitrile and alkylated with iodoacetamide (12.5 mM) in ammonium bicarbonate buffer (50 mM) for 45 min at room temperature in the dark. The samples were then digested with trypsin overnight at 37°C and the digestion was halted with 10% trifluoroacetic acid. The digested peptides were extracted twice with a 50% acetonitrile aqueous solution containing 1% formic acid, and then the volatile matter was removed using a SpeedVac. The remaining sample was dissolved in 20 µL of water containing 0.1% formic acid.

For LC-MS/MS analysis, the obtained peptide mixtures were loaded onto a trapping column and flushed using a reverse-phase analytical column with a Dionex Ultimate 3000 HPLC system (Thermo) connected to an Orbitrap Fusion Lumos mass spectrometer (Thermo). The sample was separated with solvent A (0.1% formic acid) and solvent B (100% acetonitrile and 0.1% formic acid) over 135 min. The mass spectrometer was operated in data-dependent mode, and the MS1 spectra were acquired by the Orbitrap at a target mass range of 300 to 1500 m/z with a resolution of 120,000. The 20 most intense ions were isolated for fragmentation in the higher energy collision-induced dissociation (HCD) collision cell used in MS2 scans, with a dynamic exclusion of 15 s and a 0.7 Da isolation window, normalized to 35% collision energy. The automatic gain control (AGC) target was 20,000, with a maximum injection time of 60 ms.

The raw MS data were analyzed with Proteome Discoverer software (version 2.1, Thermo), and the generated MS/MS spectra were searched against the mouse database using the Sequest HT algorithm in Proteome Discoverer software (PD, version 1.4). The database search parameters were as follows: fixed modifications of TMT 6-plexon lysine or the peptide N-terminus and carbamidomethylation on cysteine; variable modification of oxidation on methionine; two trypsin missed cleavages were permitted; the tolerances of precursor and fragment mass were 10 ppm and 0.02 Da, respectively; at least two unique peptides were used for identification of proteins; and a 1% FDR (false discovery rate) at the peptide-spectrum match (PSM) level.

**Single-cell RNA sequencing**

B16F10 tumor-bearing mice were treated with SUP3 and PBS as described above. There were 10 mice in each group, and single-cell suspensions of tumor tissue were obtained. Cells were blocked with anti-CD16/32 Abs and stained with flow-through antibodies against CD45, CD3, CD19, CD11c, NK1.1, and CD11b. Flow cytometry was used to sort CD45− nonimmune cells, CD45^+^CD3^+^ T cells, CD45^+^CD19^+^ B cells, CD45^+^CD3^−^CD19^−^CD11c^+^ DCs, CD45^+^CD3^−^CD19^−^CD11c^−^NK1.1^+^ cells and CD45^+^CD3^−^ CD19^−^CD11c^−^NK1.1^−^CD11b^+^ cells. Since it is not necessary to use single-cell data to detect the proportion of tumor-infiltrating cells, the abovementioned sorted cells were mixed into one sample for single-cell sequencing. The following preparation steps were performed before sample loading: the sample volume was controlled (>60 μl), the sample concentration was adjusted (700-1200 cells/μl), the cell clumping rate was <15%, and the cell diameter was <30 μm. Cell viability (>90%) was detected on a Countstar Rigel S2 instrument using AO/PI dual-fluorescence dye. After the cells were qualified, they were further purified using a Defragmentation Kit (MACS) and a Kill Cell Kit (MACS). The cells that passed quality control were subjected to library construction and quality control according to the "ChromiumNextGEMSingleCell3_v3.1_Rev_D" SOP of 10x Genomics. First, a single-cell suspension was generated in the Chromium Controller. Gel beads, in which reagents were encapsulated into oil droplets, were used to form GEMs. Then, in the oil droplet, the cells were lysed to release RNA, which bound to the poly(dT) primer of the tagged UMI to carry out extension of the complementary strand and addition of CCC at the end of the extension, which can pair with the rGrGrG sequence of TSO. Reverse transcription was accomplished by extension using TSO as a template. Next, recovery, amplification and quality inspection of cDNA were performed, the GEMs were broken, and magnetic beads were used to recover cDNA, amplify the full-length cDNA by PCR, and purify the amplified cDNA. Quality control of cDNA was performed with qubit 4.0 and cDNA integrity measurements (Agilent 2100). An appropriate amount of cDNA was selected for library construction, including end repair, fragmentation, A-tailing, adapter ligation and index amplification. At the same time, quality control of the library concentration (qubit4.0) and quality control of the library fragments (Qseq400) were performed. Finally, the libraries that passed the quality inspection were sequenced with a sequencer (Illumina NovaSeq 6000 PE150), and the obtained data were used for subsequent analysis.
